# Carbon Nanomaterial-Based Electrochemical Biosensors for Alzheimer’s Disease Biomarkers: Progress, Challenges, and Future Perspectives

**DOI:** 10.3390/bios15100684

**Published:** 2025-10-09

**Authors:** Berfin Şak, Helena B. A. Sousa, João A. V. Prior

**Affiliations:** LAQV, REQUIMTE, Laboratory of Applied Chemistry, Department of Chemical Sciences, Faculty of Pharmacy, University of Porto, 4050-313 Porto, Portugal; bsak@ff.up.pt (B.Ş.); up201608499@ff.up.pt (H.B.A.S.)

**Keywords:** antibody, amyloid-beta, aptamer, carbon dots, carbon nanotube, carbon nanomaterial, electrochemical biosensor, graphene, graphitic carbon nitride, human serum, molecularly imprinted polymer, point-of-care, tau protein

## Abstract

Alzheimer’s disease (AD) requires early and accurate identification of affected brain regions, which can be achieved through the detection of specific biomarkers to enable timely intervention. Carbon nanomaterials (CNMs), including graphene derivatives, carbon nanotubes, graphitic carbon nitride, carbon black, fullerenes, and carbon dots, offer high conductivity, large electroactive surface area, and versatile surface chemistry that enhance biosensor performance. While such properties benefit a wide range of transduction principles (e.g., electrochemical, optical, and plasmonic), this review focuses on their role in electrochemical biosensors. This review summarizes CNM-based electrochemical platforms reported from 2020 to mid-2025, employing aptamers, antibodies, and molecularly imprinted polymers for AD biomarker detection. Covered topics include fabrication strategies, transduction formats, analytical performance in complex matrices, and validation. Reported devices achieve limits of detection from the femtomolar to picogram per milliliter range, with linear ranges typically spanning 2–3 orders of magnitude (e.g., from femtomolar to picomolar, or from picogram to nanogram per milliliter levels). They exhibit high selectivity against common interferents such as BSA, glucose, uric acid, ascorbic acid, dopamine, and non-target peptides, along with growing capabilities for multiplexing and portable operation. Remaining challenges include complex fabrication, limited long-term stability and reproducibility data, scarce clinical cohort testing, and sustainability issues. Opportunities for scalable production and integration into point-of-care workflows are outlined.

## 1. Introduction

In 2021, an estimated 57 million people were living with dementia worldwide, over 60% of whom reside in low- and middle-income countries, with nearly 10 million new cases occurring each year [1,2]. Alzheimer’s disease (AD) is the most widespread neurodegenerative disorder and the leading cause of dementia, impacting about 30 million individuals globally, with projections indicating that this figure will increase over the next 20 years [3,4]. It is characterized by progressive decline in brain function, first evidenced in the entorhinal cortex and hippocampus, which are regions critically involved in memory formation [5]. Pathological alterations in these regions are driven by the formation of abundant plaques and tangles [6]. The plaques consist of extracellular deposits of amyloid-beta (Aβ) peptide, while the tangles are formed by abnormally phosphorylated tau proteins. In the context of Alzheimer’s disease, amyloid-beta (Aβ) and tau proteins are considered endogenous biomarkers, that is, body-inherent substances whose altered abundance, modification state, or aggregation reflects pathological processes. Such biomarkers differ from exogenous markers, which are artificially introduced probes used in imaging or spectroscopy, for example, fluorescent dyes, Raman-active tags, or radiotracers. In clinical practice, endogenous biomarkers are most commonly detected through cerebrospinal fluid (CSF) and blood serum analyses [5]. Establishing a definitive diagnosis of AD in living patients remains challenging, requiring specific biomarkers in combination with comprehensive clinical assessments. Traditional diagnostic methods can involve clinical evaluation, cognitive and neuropsychological testing, neuroimaging, biomarker analysis, genetic testing, and laboratory investigations. In current practice, diagnosis most often relies on a combination of Magnetic Resonance Imaging (MRI) to detect structural brain changes, Positron Emission Tomography (PET) to assess amyloid or tau deposition in selected cases, CSF analysis, and neuropsychological evaluation [7]. Although crucial, these methods are costly, time-consuming, and often limited in accessibility, leading to late or missed diagnoses. Early detection is essential, as there is no treatment capable of halting or reversing disease progression once clinical symptoms appear. Evidence suggests that diagnosing AD in the prodromal or mild cognitive impairment stage can reduce progression to dementia by about one-third, mainly through earlier intervention and better management of modifiable risk factors [6]. To complement this overview, Table 1 summarizes the main diagnostic method classes for AD, including representative techniques, benefits, and drawbacks.

Recent advances in blood-based biomarker testing offer promising, less invasive alternatives to traditional methods. A key development is the first FDA-authorized blood-based in vitro diagnostic device for detecting amyloid pathology in adults aged 55 and older with cognitive symptoms. The Lumipulse^®^ G pTau217/β-amyloid1-42 Plasma Assay (Fujirebio Diagnostics, Inc., Tokyo, Japan), authorized in 2025, measures the pTau217/Aβ_1–42_ ratio using chemiluminescent enzyme immunoassay (CLEIA) on the Lumipulse G1200 analyzer. In a study involving ~500 participants, the assay achieved ~92% sensitivity and 97% specificity for amyloid plaque detection compared with PET or CSF analysis [8]. It received FDA 510 (k) clearance and Breakthrough Device designation, aiming to support earlier diagnosis and treatment planning in specialized clinical settings [9].

Although highly accurate, this platform is currently restricted to specialized laboratories, fueling interest in developing other biosensor technologies, particularly portable, cost-effective, point-of-care (POC) devices, to enable broader access to early AD diagnosis. Compared with traditional diagnostic approaches, which are often complex, expensive, invasive, and time-consuming, biosensors offer a promising route to rapid, sensitive, and selective biomarker detection. By enabling earlier intervention, these technologies have the potential to slow disease progression, improve patient outcomes, and enhance quality of life [12].

In early AD detection, analytical methods such as spectroscopy and chromatography can provide multiplexed analysis [10], and techniques like Raman spectroscopy have been explored for rapid, non-destructive biomarker detection [6,11]. However, their low selectivity in complex matrices, high operational costs, and requirement for specialized equipment and expertise limit widespread use. In contrast, electrochemical biosensors combine high sensitivity, accuracy, selectivity, and cost-effectiveness, with compatibility for miniaturization and application to complex fluids such as blood or CSF [13]. The most common electrochemical methods for AD biomarker detection include differential pulse voltammetry (DPV), square wave voltammetry (SWV), cyclic voltammetry (CV), and electrochemical impedance spectroscopy (EIS) [14].

Electrochemical biosensors for AD incorporate three main classes of recognition elements: aptamers, antibodies, and molecularly imprinted polymers (MIPs). Antibodies are the most traditional recognition elements, offering high affinity and specificity in immunodiagnostic assays, though their use can be limited by high production costs, batch-to-batch variability, and reduced stability under storage or operating conditions. In contrast, MIPs provide fully synthetic, robust, and cost-effective alternatives with good reproducibility in complex matrices, albeit often with lower binding affinities than biological receptors. As extracellular amyloid plaques are a hallmark of AD, aptamers have been extensively investigated as molecular recognition elements for their early detection [10]. Aptamers are synthetic single-stranded oligonucleotides that bind selectively to specific targets via defined three-dimensional structures, offering high sensitivity in bioanalytical assays. When integrated into electrochemical devices, they form aptasensors, a promising class of POC diagnostic platforms. However, the intrinsic non-conductive nature of aptamers limits electron transfer, which can negatively affect electrical performance. The direct immobilization of aptamers onto bare electrode surfaces is often inefficient due to the limited number of binding sites, difficulty in forming uniform monolayers, and restricted analyte accessibility, all of which reduce sensitivity and reproducibility [15].

To overcome these challenges, conductive or semi-conductive nanomaterials such as gold nanoparticles, graphene, and carbon nanotubes are widely employed. These nanomaterials enhance electron transfer, provide a large surface area for aptamer immobilization, and improve overall sensor performance through signal amplification and stable probe anchoring [16]. Amongst them, carbon nanomaterials (CNMs), including graphene, carbon nanotubes, and carbon nanodots, offer exceptional advantages for electrochemical biosensors. Their excellent electrical conductivity facilitates efficient electron transfer between redox-active species and the electrode surface, a feature especially valuable when redox-active tags (e.g., ferrocene or methylene blue) are used to generate measurable electrochemical signals upon target binding [17]. CNMs also possess abundant surface functional groups such as hydroxyl and carboxyl moieties, which can be further tailored through chemical treatments to enhance bioreceptor immobilization. Moreover, they increase the electrode’s effective surface area, enabling higher immobilization densities, and can be engineered in terms of charge, shape, and size to optimize sensor performance [18,19]. Nonetheless, several challenges have been reported. CNTs tend to agglomerate due to strong van der Waals interactions, which hampers their dispersion and leads to poor interfacial contact with the electrode surface or polymer matrix [20]. This often results in the formation of heterogeneous or non-uniform films, especially when simple drop-casting or dip-coating methods are employed, where coffee-ring effects and uneven drying can produce irregular surface coverage [21]. Such structural inhomogeneities not only reduce the effective electroactive surface area but also contribute to significant reproducibility issues, since electrode performance becomes highly dependent on local film morphology and preparation conditions [21]. Eres et al. (2010) [22] emphasized that achieving stable, homogeneous, and reproducible CNT-based layers requires careful optimization of dispersion techniques, surface functionalization, and deposition methods. Furthermore, while CNMs are often assumed to be intrinsically electrocatalytic, recent single-entity measurements and mechanistic studies suggest that their apparent activity may also arise from mass-transport effects, thin-layer diffusion, or residual impurities, rather than from true catalytic behavior [23,24]. These complexities highlight the importance of cautious interpretation of their electrochemical properties and of adopting strategies to improve film homogeneity and reproducibility.

Considering their high redox activity, versatile functionalization potential, exceptional electrical conductivity, compatibility with biochemical receptors, and ability to integrate with auxiliary reagents, CNMs are exceptionally well-suited for the construction of electrochemical biosensors employing aptamers, antibodies, or MIPs. This review discusses recent advances in CNM-based electrochemical biosensors for AD biomarker detection, focusing on developments reported between 2020 and mid-2025, and addressing analytical performance, validation in complex matrices, and challenges toward clinical translation.

## 2. Carbon Nanomaterials

Nanomaterials (NMs) are an extraordinary category of materials with any external dimension between 1 and 100 nanometers or with internal or surface structures in that range [25,26]. Owing to their nanoscale dimensions, they typically exhibit a high surface area to volume ratio compared with their bulk counterparts, a property that enhances their reactivity and interaction with surrounding media. NMs can display unique magnetic, electrical, optical, mechanical, and catalytic properties that differ markedly from those of larger, bulk materials. These properties can be tailored by precisely controlling parameters such as size, shape, synthesis conditions, and surface functionalization [27,28].

Carbon nanomaterials (CNMs), which are allotropic forms of carbon at the nanoscale, were first identified with the discovery of Buckminster fullerene in 1985 [29]. Over the past two decades, extensive research has established CNMs as one of the most widely used classes of nanomaterials. Their remarkable optical, mechanical, and electrical properties have led to applications across diverse industrial, technological, and biomedical fields. Several intrinsic characteristics make CNMs particularly well-suited for electrochemical sensor (ECS) applications. These include their ability to increase the electroactive surface area, enabling higher immobilization densities; their excellent electrical conductivity, which enhances electron transfer between redox active species and the electrode surface; and their surface functional groups (SFGs, e.g., hydroxyl and carboxyl), which promote molecular adsorption and can be chemically modified to tune charge, shape, and size. CNMs have been integrated into ECS designs for biomolecule detection using various deposition techniques, most commonly dip coating and drop casting [17,18,30].

**Figure 1:**
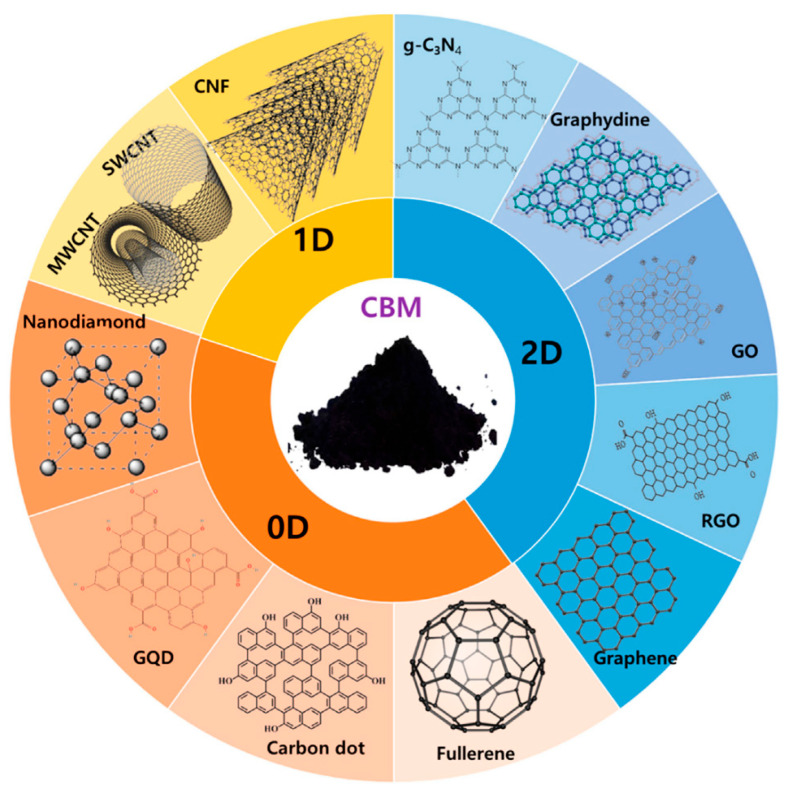
Classification of selected carbon nanomaterials by dimensional properties, including carbon dots, nanodiamonds, fullerenes, nanotubes, and graphene. CBM: Carbon-based Nanomaterial; GQD: Graphene Quantum Dot; GO: Graphene Oxide; RGO: Reduced Graphene Oxide; SWCNT: Single Walled Carbon Nanotube; MWCNT: Multi Walled Carbon Nanotube; CNF: Carbon Nanofiber; g-C_3_N_4_: graphitic carbon nitride. Reproduced from Mohapatra et al. (2023) [35], under CC BY 4.0.

Outside of SFGs, the interfacial behavior of CNMs is controlled by texture (specific surface area and porosity), crystallographic orientation (basal vs. edge sites), as well as defect classes (e.g., vacancies, Stone-Wales rearrangements or heteroatom doping). These factors affect (or modulate) charge transfer, adsorption energetics and film uniformity as well as the resulting analytical performance of electrochemical biosensors. A comprehensive discussion of CNM surface physics and defect chemistry is beyond the scope of this review and can be found in specialized works such as the Handbook of Carbon Nanotubes (Springer, 2022) [31]. Here, we focus only on the relevant implications for sensor interfaces, namely electron transfer pathways, immobilization density, and interfacial reproducibility, while referring to SFGs as the chemical functionalities that support biomolecule anchoring and interfacial tuning in biosensor design. CNMs can be categorized according to their dimensional properties, ranging from zero-dimensional (0D) to one-dimensional (1D) and two-dimensional (2D) structures, as defined by their nanoscale dimensions in different spatial orientations (Figure 1) [30,32]. We note that CNMs can also form three-dimensional assemblies, such as buckypapers, CNT yarns, or random aggregates, which are reviewed elsewhere [33,34]. However, these architectures are not discussed here, as the present review focuses on 0D, 1D, and 2D CNMs in electrochemical biosensing. In this review, CNMs are grouped into six sub-categories, following the classification proposed in [10]: graphene-based nanomaterials, carbon nanotubes (CNTs), graphitic carbon nitride (g-C_3_N_4_), carbon black (CB), fullerenes, and carbon dots (CDs). In the following sections, for each CNM category, we consider both pristine materials and CNM-based composites whenever relevant to electrochemical sensing performance.

### 2.1. Graphene-Based Nanomaterials

Graphene, a two-dimensional (2D) allotrope of carbon discovered by Novoselov et al. in 2004 [36], occurs in various nanostructured forms differing in the number of layers but consistently exhibiting a honeycomb lattice of sp^2^-hybridized carbon atoms [37]. It is an exceptional thermal conductor, with reported thermal conductivity values up to ~5300 W m^−1^ K^−1^ [38], is highly impermeable to gases [39], readily functionalized [40], and possesses a large specific surface area (~2630 m^2^ g^−1^) [41].

Graphene synthesis can be broadly divided into top-down and bottom-up approaches. The top-down approach starts from graphite, with graphene isolated via exfoliation methods that may be chemical, mechanical, or thermal. Micromechanical exfoliation, the so-called “Scotch tape method”, uses adhesive tape to peel graphene flakes from graphite [36], while liquid-phase exfoliation disperses graphite in solvents followed by sonication to obtain graphene sheets [42]. Thermal exfoliation of graphite oxide involves rapid heating, generating high internal gas pressure that causes exfoliation into graphene [43]. In contrast, bottom-up synthesis forms graphene from carbon precursors at high temperatures, enabling controlled growth of large area, high-quality films, suitable for electronic applications [44]. Two widely used methods are chemical vapor deposition (CVD), where graphene is grown on metal substrates such as copper [45], and epitaxial growth on silicon carbide (SiC), in which thermal decomposition of SiC produces graphene layers [46].

Graphene and its derivatives, particularly graphene oxide (GO) and reduced graphene oxide (rGO), are promising for industrial, environmental, and biomedical applications owing to advances in synthesis and functionalization. Scalable methods such as CVD produce high-quality films for electronics [45], while the improved Hummers method, using KMnO_4_ and a 9:1 mixture of concentrated H_2_SO_4_/H_3_PO_4_, facilitates GO production for composites and drug delivery [47]. Functionalization improves surface properties, enabling applications such as contaminant removal [48] and targeted biomedical uses including drug delivery and photothermal therapy [49].

GO, an oxygen-rich derivative of graphene, contains abundant functional groups such as epoxides, hydroxyls, carbonyls, and carboxylates, and demonstrates superior performance, particularly in biosensing applications, compared with other graphene forms. This enhanced performance is largely attributed to its oxygen-containing groups, which improve water dispersibility [48], enable efficient biomolecule immobilization [50], and enhance analyte interaction and electron transfer during detection [51,52]. GO’s hydrophilic nature contrasts with the hydrophobicity of pristine graphene, further facilitating its integration into aqueous sensing systems. Structurally, GO consists of both aliphatic (sp^3^) and aromatic (sp^2^) domains [53]: the sp^3^ regions introduce defects and host oxygen functionalities that promote molecular adsorption, while the sp^2^ regions provide conjugated, conductive pathways that support electron transfer and strong π–π interactions. This combination of chemical versatility and electronic conductivity makes GO more functionally adaptable than pure sp^2^ carbon materials such as pristine graphene [54,55].

**Figure 2:**
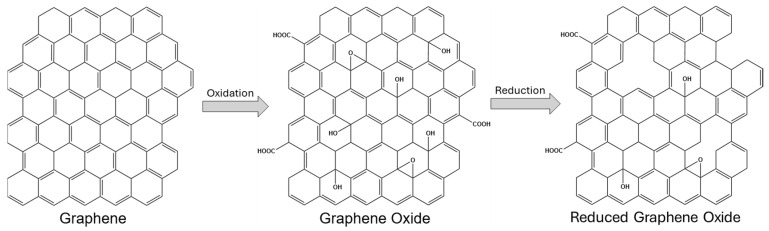
Chemical structures and synthesis methods of graphene, graphene oxide, and reduced graphene oxide. Adapted from Bellier et al. (2022) [56], under CC BY 4.0.

Although GO is electrically insulating due to the disruption of its sp^2^ bonding network, its conductivity can be partially restored by reduction processes that re-establish π conjugation. The material obtained through this process is commonly referred to as reduced graphene oxide (rGO) [48]. Depending on the reduction method, rGO retains varying amounts of oxygen-containing groups, which can be advantageous for certain applications, as they preserve sites for further functionalization while improving electrical conductivity. The structural differences between graphene, GO, and rGO, along with their typical structures resulting from redox reactions involved in synthesis, are illustrated in Figure 2.

Graphene-based composites, including those incorporating conductive polymers, metal matrices, carbon-carbon matrices, and natural fibers, exhibit high mechanical strength, electrical conductivity, and thermal stability, making them attractive for applications such as energy harvesting, clean energy storage, and wearable or portable electronics [57]. In addition, graphene-based nanomaterials have been extensively used in biosensor development, where they enable sensitive and selective detection of disease biomarkers [53]. Representative graphene-based biosensing examples are detailed in Section 3 alongside other CNM categories for consistency.

### 2.2. Carbon Nanotubes

Carbon nanotubes (CNTs), discovered by Iijima in 1991 [58,59], are valued for their exceptional mechanical, thermal, and electrical properties, as well as their extremely high aspect ratios (length to diameter ratios). These tubular carbon structures, resembling rolled graphene sheets composed of hexagonally arranged sp^2^ hybridized carbon atoms, stand out among other carbon-based nanomaterials (CNMs) such as graphene, nanodiamonds, and carbon nanofibers [60]. Properties that distinguish CNTs from other CNMs include outstanding mechanical flexibility [61], quasi-ballistic electron transport [62], and their ability to be metallic or semiconducting depending on chirality and diameter [63]. This tunability makes CNTs highly attractive for applications in nanoelectronics, biosensing, drug delivery, and energy storage [64,65].

The structure of CNTs is defined by three key parameters: number of walls, chirality, and length. Chirality determines the CNT’s diameter and strongly influences its electronic behavior. Single-walled CNTs (SWCNTs) consist of a single graphene sheet rolled into a cylinder, while multi-walled CNTs (MWCNTs) contain multiple concentric cylinders. Double-walled CNTs (DWCNTs), the simplest form of MWCNTs, are composed of two concentric SWCNTs held together by weak van der Waals forces. Typical SWCNT diameters range from 0.4 nm to 3 nm, whereas MWCNT diameters vary from ~2 nm to over 100 nm depending on synthesis conditions. Due to their narrow diameters and extended tubular morphology, CNTs can achieve aspect ratios exceeding 1000 and surface areas greater than 1300 m^2^ g^−1^ [66].

CNTs are typically synthesized by arc discharge [59], laser ablation [67], or chemical vapor deposition (CVD) [68]. Arc discharge, one of the earliest techniques, involves vaporizing carbon rods in a reduced pressure and inert atmosphere, requiring a metal catalyst for SWCNT growth [69]. Laser ablation, developed by Smalley’s group in 1995, uses a pulsed laser to vaporize graphite in a helium-filled chamber, achieving yields of up to 70% [70]. However, both arc discharge and laser ablation require high temperatures and are limited in scalability. CVD has largely replaced them for large-scale production, decomposing gaseous carbon sources (e.g., methane) at 650–900 °C on catalyst-coated substrates, with yields of around 30% [70,71].

The surface of CNTs consists of sp^2^ hybridized carbon atoms, where three valence electrons form σ bonds and the fourth occupies a p orbital perpendicular to the tube wall. In planar graphene, these p orbitals overlap to form π and π* bands, giving a semi metallic character. Rolling graphene into a cylinder induces curvature, leading to π–σ hybridization and one-dimensional (1D) quantum confinement, where electrons are restricted in the radial and circumferential directions and can only move freely along the nanotube axis. This quantizes their energy levels into discrete subbands [72]. The resulting electronic properties, including high carrier mobility, a tunable bandgap in semiconducting SWCNTs, and ballistic transport in metallic SWCNTs, are governed by the nanotube’s diameter, chirality, and wall number, and are critical for applications in electronics, chemical sensing, and biosensing [73,74].

**Figure 3:**
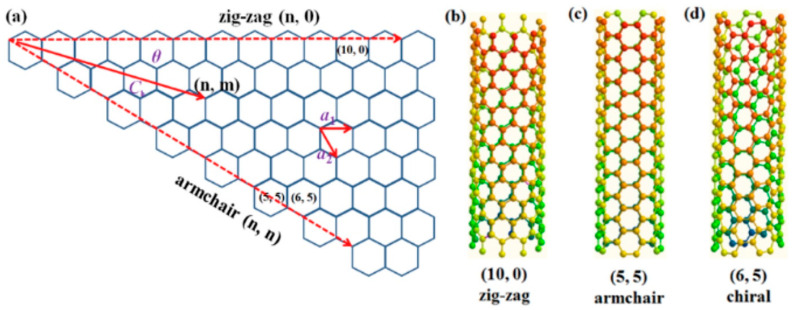
Single-walled carbon nanotube (SWCNT) structures: (**a**) unrolled zigzag graphene sheet showing tube geometry and chirality angle (θ); (**b**) zigzag, (**c**), and (**d**) chiral sidewall configurations. Reprinted from Zhang et al. (2016) [77], Copyright (2016), with permission from Elsevier.

The C–C bond length increases slightly from around 1.42 Å in graphene to ~1.44 Å in SWCNTs, due to curvature. The curvature results from rolling the graphene sheet into a cylinder, while the rolling angle determines the chirality of the CNT. Hence, chirality is defined by the chiral vector (Ch = na1 + ma2), which thus describes how the graphene sheet is rolled. In Ch, the integer indices n and m (n, m) are the number of steps alongside the a1 and a2 unit vectors [75], and the a1 and a2 are the basis vectors of the hexagonal unit cell, which connect two crystallographically equivalent sites on the graphene lattice. The chirality angle (θ) is calculated as θ = tan^−1^[√3m/(2n + m)], lies between 0° and 30°, and together with (n, m), differentiates the CNTs in three types: when θ = 0° (m = 0) corresponds to zigzag geometry configuration (n, 0); whilst when θ = 30° (m = n) to armchair configuration (n, n); the other types, when 0° < θ < 30°, are called chiral (n, m) (Figure 3). Additionally, CNTs show metallic and semiconducting properties depending on the chirality (n, m) [76]: they are metallic when n = m (armchair) or when (n − m) is a multiple of 3 (n − m = 3 k); otherwise, they are semiconducting materials.

MWCNTs can exhibit different morphologies influenced by rolling defects in the graphene layers. Common forms include hollow tubes (parallel graphene walls), herringbone structures (angled walls), and bamboo-like structures (periodic internal compartments). Defects in MWCNT walls often introduce oxygen-containing functional groups, which can facilitate redox catalysis and improve electron transfer in electrochemical applications [78]. CNTs have also been widely integrated into electrochemical biosensors for AD biomarkers. For example, Kim et al. (2020) [6] reported a CNT-based composite platform that enabled highly sensitive detection of Aβ peptides and tau proteins, demonstrating improved signal amplification and enabling the detection of much lower biomarker concentrations than was possible with bare electrodes.

### 2.3. Graphitic Carbon Nitride

Carbon nitride refers to compounds composed of carbon and nitrogen atoms, with early studies dating back to 1816 following the discovery of paracyanogen [79]. Interest in this class of materials intensified after Liu and Cohen [80], in 1989, predicted the potential synthesis of superhard carbon nitride phases. Earlier, in 1979, the Cuomo group [81] attempted the synthesis of polymeric carbon nitride, proposing a planar, layered structure. Since then, several allotropes have been reported, among which graphitic carbon nitride (g-C_3_N_4_) is the most stable and extensively studied. Unlike other carbon nitride phases, g-C_3_N_4_ exhibits sp^2^ hybridization for both carbon and nitrogen atoms [82,83].

**Figure 4:**
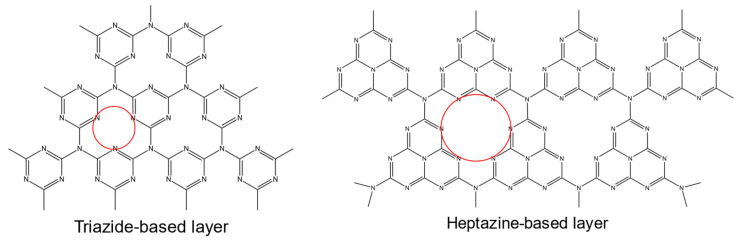
Proposed planar configurations of C_3_N_4_ (g-C_3_N_4_). Highlighted in red circles are the typical intrinsic vacancy sites, each surrounded by nitrogen atoms. Adapted from Inagaki et al. (2022) [87], Copyright (2022), with permission from Elsevier.

The g-C_3_N_4_ structure consists primarily of two building blocks, triazine and tri s triazine (heptazine) units, which condense into a two-dimensional polymeric framework with carbon and nitrogen atoms arranged in hexagonal rings (Figure 4) [84,85]. This material demonstrates high thermal and chemical stability under both acidic and alkaline conditions, and it possesses a moderate band gap of approximately 2.7 eV, classifying it as a visible-light-active semiconductor. These properties support its widespread use in photocatalytic applications, particularly in water splitting, CO_2_ reduction, and pollutant degradation [86].

Graphitic carbon nitride can be synthesized by several techniques, including chemical vapor deposition (CVD) [88], thermal polymerization [82], and solvothermal methods [89]. Nanostructured forms of g-C_3_N_4_ exhibit enhanced properties such as increased surface area, tunable electronic structure, and improved light-harvesting ability. Beyond energy and environmental applications, these same characteristics, particularly photosensitivity, ease of surface functionalization, and biocompatibility, make g-C_3_N_4_-based nanomaterials highly attractive for biosensing. In this context, they have shown promise in the early diagnosis of Alzheimer’s disease and cancer, enabling both signal-amplified and label-free detection strategies [90,91].

### 2.4. Carbon Black

Carbon black (CB) is a nanostructured carbon material typically produced via the thermal decomposition of carbon-rich feedstocks under either an inert atmosphere (pyrolysis) or an oxygen-depleted atmosphere (partial combustion). It consists of spherical primary particles, usually 10 to 100 nm in diameter, that fuse during synthesis to form larger, branched aggregates.

The electrical properties of CB are remarkable for such a low-cost material. At 300 K, reported values for electrical conductivity, charge carrier mobility, and carrier density are approximately 2.4 S cm^−1^, 5.4 cm^2^ V^−1^ s^−1^, and 1.5 × 10^18^ g^−1^, respectively [92]. Its specific surface area can vary widely depending on synthesis conditions, ranging from 9 to 1500 m^2^ g^−1^ [93,94].

In addition to these physical properties, CB nanoparticles exhibit a high density of oxygen-containing functional groups on their surface, including hydroxyl, carbonyl, and carboxyl groups, which enable covalent immobilization of biomolecular recognition elements. Combined with their excellent electron transfer capabilities, low cost, and wide commercial availability, these characteristics make CB highly suitable for voltammetric biosensing applications, including enzymatic sensors, immunosensors, and DNA-based detection systems [95].

### 2.5. Fullerenes

**Figure 5:**
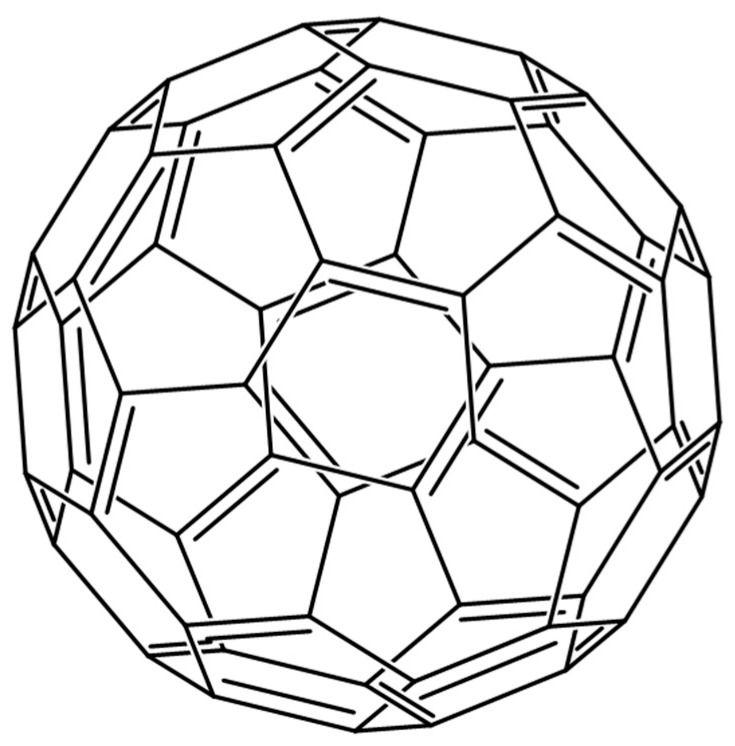
Icosahedral structure of the C60 carbon cage.

Fullerenes are a class of carbon allotropes composed entirely of sp^2^-hybridized carbon atoms arranged in closed-cage, polyhedral structures. First theorized in the early 1980s and isolated in bulk in 1990, fullerenes were discovered during studies of carbon vapor generated by laser ablation of graphite [96]. The most well-known member of this family is C60, a highly symmetrical, icosahedral molecule with a spherical π-conjugated carbon cage consisting of 12 pentagons and 20 hexagons, and a diameter of approximately 0.7 nm (Figure 5) [97].

Common synthetic methods for producing fullerenes include carbon arc discharge [98], plasma discharge [99], hydrocarbon pyrolysis [100], and laser ablation [29]. While C60 and C70 are the most abundant and widely studied forms, higher fullerenes up to C96 have also been investigated for their unique electronic and structural properties.

One particularly interesting subclass is the endohedral fullerenes, which feature encapsulated atoms or clusters within the carbon cage. These include noble gases (e.g., He, Ar, Kr, Xe) [101], diatomic molecules such as H_2_ [102], single metal atoms like La, Sc, and Y [103], or complex clusters such as Sc_3_N@C_80_ and YCN@C_82_ [104]. Encapsulation can alter the electronic properties of the fullerene host, making these structures attractive for a wide range of applications.

Fullerenes can interact non-covalently with other carbon nanomaterials, such as carbon nanotubes (CNTs) and graphene, through hydrophobic interactions and π–π stacking [105]. These interactions can enhance electron transfer and electrocatalytic performance in electrochemical sensors. For example, a fullerene–MWCNT composite has been used to detect vanillin in real food samples [106], and a fullerene–graphene oxide (GO) composite has been employed for the detection of dopamine in rat brain tissue and pharmaceutical injection samples [107].

Beyond sensing, fullerenes and their derivatives are utilized in molecular electronics, photovoltaics, and biomedical fields, including drug delivery, antiviral therapies, imaging, and photodynamic therapy, due to their versatile electronic structure, stability, and biocompatibility. These diverse applications underscore their continued relevance and value in scientific research [108].

### 2.6. Carbon Dots

Carbon dots (CDs) are a class of fluorescent, zero-dimensional carbon nanomaterials composed of spherical particles typically smaller than 10 nm. First reported by Xu et al. in 2004 [109], CDs have attracted considerable interest due to their distinctive properties, including strong and tunable photoluminescence, excellent water solubility, high biocompatibility, low toxicity, robust photostability, notable catalytic activity, and facile surface functionalization [110]. These features make CDs promising candidates for a wide range of applications, such as sensing, imaging, catalysis, medicine, and optoelectronics. In particular, their favorable toxicity profile and optical characteristics position them as environmentally friendly alternatives to semiconductor quantum dots (QDs), placing them at the vanguard of current nanotechnology research [111].

CDs were typically classified into three categories, carbon nanodots (CNDs), carbon quantum dots (CQDs), and graphene quantum dots (GQDs), based on their structural order and optical behavior [112]. But, very recently, carbonized polymer dots (CPDs) have been recognized as a new category of CDs [113]. CNDs are amorphous or quasi-spherical nanoparticles that lack long-range crystallinity and do not exhibit quantum confinement. In contrast, CQDs are nearly spherical, crystalline carbon nanostructures smaller than 10 nm that display quantum confinement effects and size-dependent fluorescence. GQDs consist of small graphene fragments with lateral dimensions below 100 nm and a few atomic layers in thickness. These structures combine the quantum effects observed in CQDs with the intrinsic electronic and optical features of graphene, resulting in materials with high surface-to-volume ratios, long fluorescence lifetimes, and strong resistance to photobleaching [114]. CPDs differ from these categories by featuring a polymer/carbon hybrid structure formed through incomplete carbonization of polymer clusters, which provides many surface functional groups, high photoluminescence quantum yields, and unique emission mechanisms such as molecular state and crosslink-enhanced emission.

The physicochemical characteristics of CDs, such as size, crystallinity, surface chemistry, and emission behavior, depend heavily on the synthesis method and the nature of the precursors used. Generally, two broad approaches are employed for CD synthesis: top-down and bottom-up strategies. Top-down methods involve the physical or chemical breakdown of bulk carbon-rich materials, including graphite, graphene, fullerenes, soot, carbon nanotubes, activated carbon, nanodiamonds, and even organic waste sources like biomass or food residues. These materials are reduced to nanoscale dimensions through techniques such as laser ablation, arc discharge, electrochemical oxidation, chemical oxidation, or ultrasonication [115]. Bottom-up methods, on the other hand, involve the carbonization of molecular precursors such as carbohydrates, organic acids, natural products, and polymers. These precursors are transformed into carbon nanostructures using processes like hydrothermal or solvothermal treatment, pyrolysis, thermal decomposition, microwave irradiation, or direct carbonization [116].

Importantly, the choice of synthetic route significantly influences the quality, size uniformity, and surface passivation of the resulting CDs. While top-down approaches often require harsh reaction conditions, extended processing times, and specialized equipment, bottom-up methods offer a more controllable, scalable, and environmentally friendly alternative for the controlled and efficient production of CDs [110]. By precisely adjusting reaction conditions, such as temperature, pH, time, and precursor concentration, researchers can tailor the optical and chemical properties of CDs to suit specific applications. This tunability, combined with their biocompatibility and cost-effectiveness, continues to support the growing interest in CDs for use in biomedical diagnostics and biosensor platforms.

## 3. Carbon-Based Electrochemical Sensors for Alzheimer’s Disease Biomarker Detection

Electrochemical sensors, renowned for their excellent sensitivity and selectivity, are highly effective analytical devices for detecting a broad range of analytes. In biosensing, a suitable molecular recognition element interacts with the target analyte at the sensor surface, producing a measurable electrochemical response through an integrated transducer. These platforms have been increasingly employed for Alzheimer’s disease (AD) biomarker detection, particularly amyloid-beta (Aβ) peptides and tau protein, using voltammetric, impedimetric, and transistor-based formats.

Electrochemical aptasensors (ECAS), first reported for protein detection using a double-aptamer sandwich configuration [117], offer high specificity and affinity for target biomarkers by employing aptamers as molecular recognition elements [5,118]. Alongside aptamers, antibodies have been widely used in electrochemical immunosensors for their established clinical specificity and affinity, while molecularly imprinted polymers (MIPs) provide a reliable, regenerable, and cost-effective synthetic alternative for selective target recognition.

This section reviews recent advances, from 2020 to mid-2025, in CNM-based electrochemical sensors for AD biomarker detection, encompassing graphene and its derivatives, carbon nanotubes, graphitic carbon nitride, and other carbon allotropes and hybrids. The discussion is organized according to CNM platform type, focusing on detection principles, analytical performance, real-sample validation, and current challenges to clinical translation. For each CNM category, both pristine materials and CNM-based composites are considered whenever relevant to analytical performance.

### 3.1. Graphene-Based Electrochemical Sensors

Graphene-based materials have emerged as leading candidates for electrochemical biosensing platforms due to their high conductivity, large specific surface area, and versatile surface chemistry. In AD diagnostics, these carbon nanostructures are often engineered with metallic nanoparticles, metal–organic frameworks, conducting polymers, or micromotor systems to improve electron transfer, catalytic activity, and biomolecule immobilization. Such hybrid configurations have enabled ultrasensitive detection of AD-related biomarkers in complex biological fluids. Table 2 compiles the main analytical performances, sensing strategies, and validation outcomes reported for graphene-based electrochemical biosensors targeting Alzheimer’s disease biomarkers.

**Figure 6:**
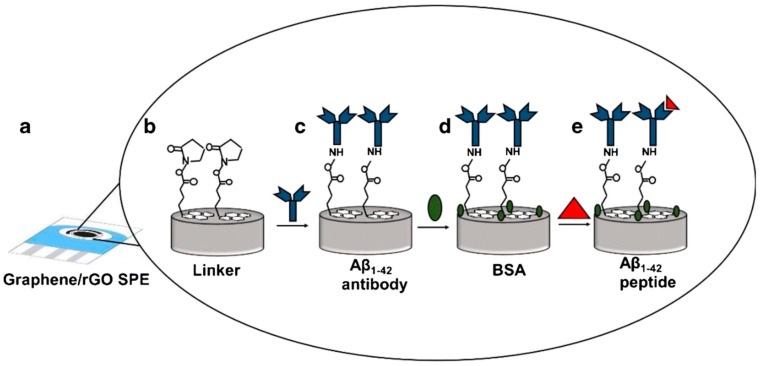
Schematic illustration of the electrochemical setup for Aβ_1–42_ detection using a graphene/rGO screen-printed electrode (**a**) sequentially modified with linker (**b**), antibody (**c**), BSA (**d**), and Aβ_1–42_ peptide (**e**). Reproduced from Sethi et al. (2020) [119], under CC BY 4.0.

A label-free electrochemical biosensor using a dual-layer architecture of graphene and reduced graphene oxide (rGO) was designed by Sethi et al. (2020) [119] for detecting the plasma biomarker Aβ_1–42_ associated with AD (Figure 6). The H31L21 antibody was immobilized via 1-pyrenebutyric acid N-hydroxysuccinimide ester (Pyr-NHS), enabling stable anchoring without compromising the rGO structure. Analytical performance was evaluated using differential pulse voltammetry (DPV), achieving a limit of detection (LOD) of 2.398 pM and a wide linear range from 11 pM to 55 nM. The biosensor exhibited high specificity towards Aβ_1–42_ over the potentially interfering species Aβ_1–40_ and ApoE ε4, even at high excess concentrations of the latter. Validation was carried out in human plasma (spiked samples) and in untreated plasma from transgenic (Tg) and wild-type (WT) mice. In human plasma, the platform displayed excellent linearity (R^2^ = 0.98) between signal and Aβ_1–42_ concentration. In the animal model, a marked difference in response was observed between Tg and WT mice, along with a decrease in plasma Aβ_1–42_ levels in 12-month-old Tg mice compared with 9-month-old Tg mice. This age-dependent decrease correlated with increased brain deposition of the peptide, confirmed by immunohistochemistry (IHC) and magnetic resonance imaging (MRI). Despite its excellent analytical performance, the authors noted limitations such as the random orientation of antibodies, which can reduce capture efficiency, and a time-consuming fabrication process due to long incubation steps. Nevertheless, the use of screen-printed electrodes (SPEs) ensures low cost, scalability, and compatibility with point-of-care integration, offering strong potential for early AD diagnosis. The authors state that future work will include larger sample sizes to assess the biosensor’s ability to discriminate between different stages of the disease.

**Figure 7:**
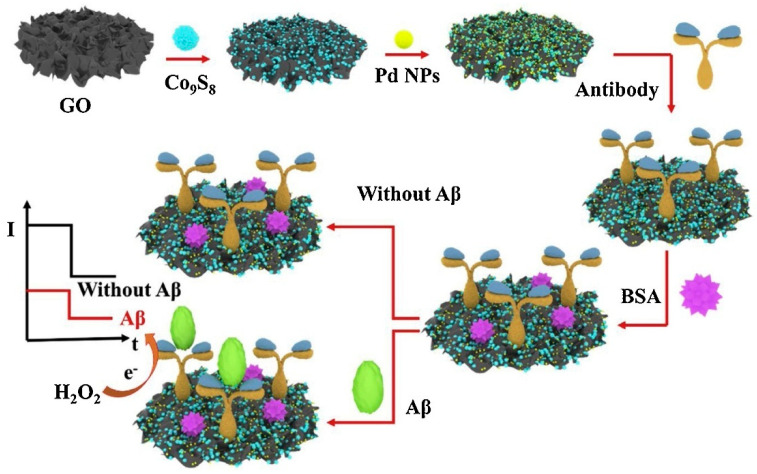
Immunosensor for Aβ detection using Pd–Co_9_S_8_/graphene oxide nanocomposite. Reprinted from Li et al. (2020) [120], Copyright (2025), with permission from Elsevier.

For Aβ detection, Li et al. (2020) [120] fabricated a label-free electrochemical immunosensor based on bifunctional Pd–Co_9_S_8_ polysulfide nanoparticles supported on graphene oxide (Figure 7). The Co_9_S_8_ nanoparticles were uniformly distributed on graphene oxide to prevent agglomeration and enhance conductivity, while Pd nanoparticles provided additional electrocatalytic activity for H_2_O_2_ reduction and enabled stable antibody immobilization through Pd–N chemical bonding. The synergistic effects among Co_9_S_8_, Pd, and graphene oxide resulted in a high specific surface area, superior conductivity, and excellent electrocatalytic performance. The analytical performance, evaluated by cyclic voltammetry (CV) and electrochemical impedance spectroscopy (EIS), showed a wide linear range from 0.1 pg/mL to 50 ng/mL and an ultralow LOD of 41.4 fM. The immunosensor demonstrated good selectivity against potential interfering species, as well as acceptable reproducibility (RSD < 5%) and stability (4.1% signal loss after three weeks at 4 °C). Practical applicability was assessed in artificial cerebrospinal fluid (CSF) using a standard addition recovery assay, achieving recoveries between 96.3% and 109.5% with RSD values of 3.9–4.9%. These results indicate the potential of the G/Co_9_S_8_–Pd-based immunosensor for clinical AD diagnosis. However, the use of Pd and Co metals warrants consideration due to possible environmental and public health impacts associated with these materials.

**Figure 8:**
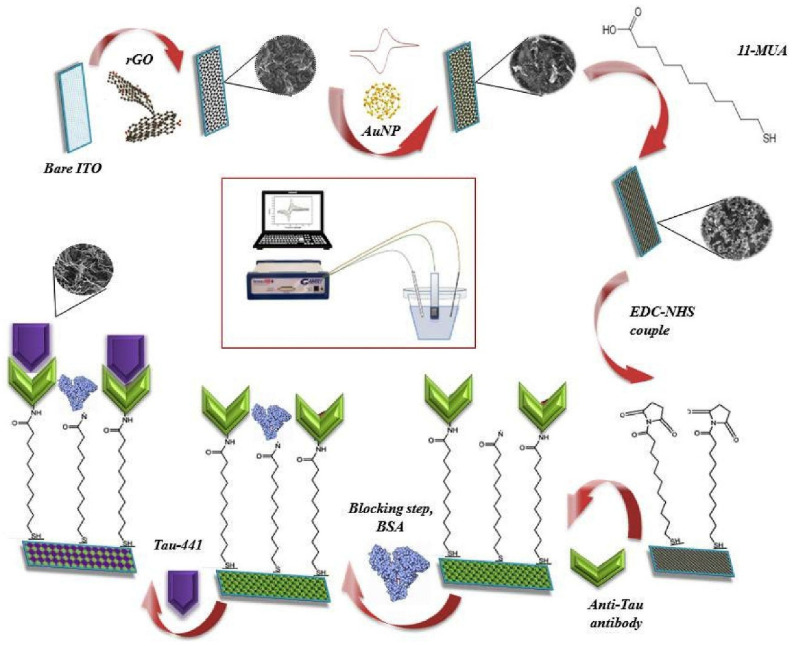
Immobilization sequence of anti-tau antibody on rGO–AuNP-modified ITO–PET electrode for Tau-441 detection. Reprinted from Karaboga and Sezgintürk (2020) [121], Copyright (2025), with permission from Elsevier.

A disposable neuro-biosensing probe targeting Tau-441 protein was proposed by Karaboga and Sezgintürk (2020) [121] as a pre-proof concept study (Figure 8). The probe was constructed on an indium tin oxide–polyethylene terephthalate (ITO–PET) substrate modified with a reduced graphene oxide–gold nanoparticle (rGO–AuNP) nanocomposite. The surface was further functionalized with 11-mercaptoundecanoic acid (11-MUA) to enable antibody immobilization. Analytical performance, assessed using cyclic voltammetry (CV) and electrochemical impedance spectroscopy (EIS), revealed an LOD of 0.091 pg/mL and a linear range from 1 to 500 pg/mL. Additionally, single-frequency impedance (SFI) measurements were employed in a non-faradaic environment to monitor the immunoreaction between Tau-441 and its specific antibody in real time. The applicability of the biosensor was demonstrated in both serum and cerebrospinal fluid (CSF) samples, with recoveries ranging from 96% to 108%. The platform offers advantages such as low cost and ease of modification due to the use of ITO–PET electrodes. However, its current preparation process is lengthy, requiring approximately two days, including incubation steps. The authors suggest that future work should focus on developing more practical fabrication methods and testing with a larger number of real clinical samples.

**Figure 9:**
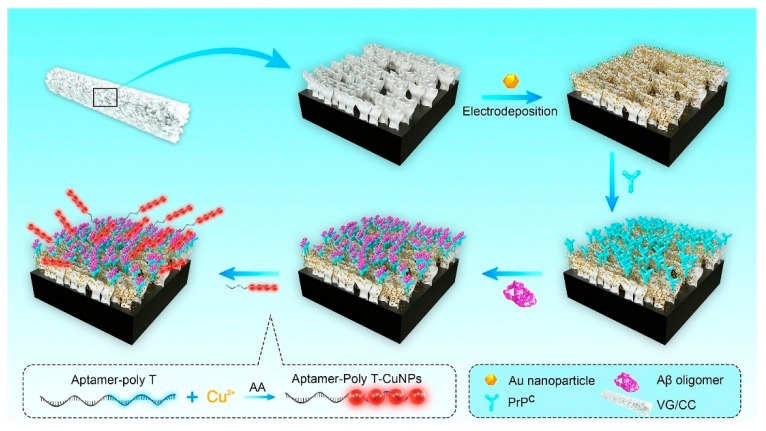
Sandwich-type electrochemical aptasensor for Aβ oligomers using AuNP-decorated vertical graphene/carbon cloth scaffold. Reprinted from Zhou et al. (2021) [122], Copyright (2025), with permission from Elsevier.

An ultrasensitive sandwich-type electrochemical aptasensor for amyloid-β oligomer (AβO) detection in human serum (Figure 9) was introduced by Zhou et al. (2021) [122], using a three-dimensional conductive scaffold composed of vertical graphene (VG) nanosheets grown directly on a flexible carbon cloth (CC) substrate and decorated with gold nanoparticles (AuNPs) (Au-VG/CC). In this architecture, the woven carbon cloth provides mechanical flexibility, electrical conductivity, and a porous three-dimensional framework, while the vertically aligned graphene sheets, synthesized on the fiber surface by plasma-enhanced chemical vapor deposition, create a high-surface-area nanostructure that enhances electron transfer and allows the uniform deposition of AuNPs. The AuNPs offer abundant active sites for immobilizing the capture recognition element, a cellular prion protein fragment (PrP^C^, residues 95–110), which selectively binds AβO. Poly(thymine)-templated copper nanoparticles (CuNPs) were synthesized in situ as signal probes to enable aptamer immobilization, establishing a dual-recognition mechanism. The biosensor assembly was characterized by cyclic voltammetry (CV), electrochemical impedance spectroscopy (EIS), scanning electron microscopy (SEM), and fluorescence spectroscopy. Differential pulse voltammetry (DPV) revealed a detection limit of 3.5 pM and a linear response range from 10 to 2200 pM in serum. The aptasensor exhibited high specificity towards AβO over five potential interferents, Aβ monomers (AβM), Aβ fibrils (AβF), tau protein, immunoglobulin G (IgG), and bovine serum albumin (BSA), good stability (retaining 89.9% of its initial activity after 4 weeks), and satisfactory repeatability (RSD 4.8%, *n* = 7) and reproducibility (RSD 6.7%, *n* = 6). Validation with human serum samples (*n* = 9) from both AD patients and healthy controls showed strong correlation with ELISA results, with recovery rates of 93–116% (RSD < 10%). Although the fabrication process involves multiple steps and the use of AuNPs raises cost and environmental considerations, the combination of vertical graphene scaffolds, noble metal decoration, and dual-recognition strategy yielded excellent analytical performance. Further simplification of fabrication and expanded clinical validation could support its translation into practical diagnostic applications.

Micromotor (MM) technology was applied by Gallo-Orive et al. (2024) [123] for the detection of Aβ oligomers-42 (AβO_42_) in complex clinical matrices, including brain tissue, cerebrospinal fluid (CSF), and plasma obtained from AD patients. MMs are micro/nanoscale devices that convert chemical or external energy into motion, often using catalytic bubble propulsion [126] or magnetic actuation [127]. Their layered designs enable propulsion, functionalization, and navigation for biomedical sensing and targeted delivery [128].

**Figure 10:**
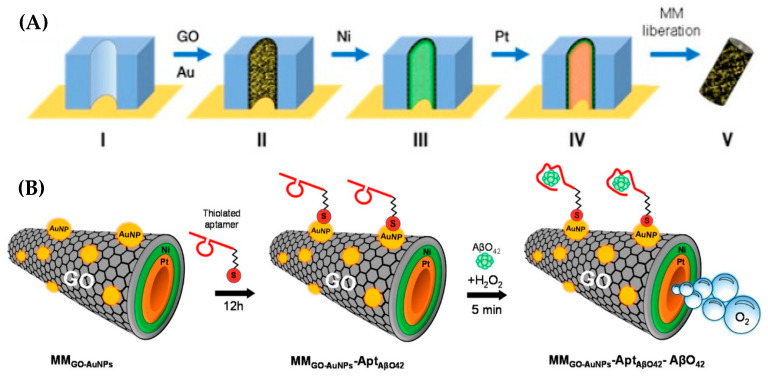
(**A**) Synthesis stages (I–V) of GO/AuNP/Ni/PtNP micromotors (MM_GO–AuNPs_): (I) pristine membrane serving as the structural template; (II) deposition of a graphene oxide layer decorated with gold nanoparticles (GO–AuNPs), providing the outer surface for aptamer attachment; (III) addition of a nickel layer that imparts magnetic responsiveness and assists in the washing steps of the assay; (IV) incorporation of an inner platinum nanoparticle layer acting as a catalyst for oxygen bubble propulsion in hydrogen peroxide medium; and (V) removal of the membrane template, yielding free micromotors ready for surface biofunctionalization. (**B**) Functionalization of MM_GO–AuNPs_ with thiolated aptamer for AβO_42_ recognition and on-the-fly aptassay for AβO_42_ detection (MM_GO–AuNPs_–Apt_AβO42_–AβO_42_). Adapted from Gallo-Orive et al. (2024) [123], under CC BY 4.0.

The developed micromotor platform was based on graphene oxide/gold nanoparticles/nickel/platinum nanoparticles (GO/AuNPs/Ni/PtNPs, Figure 10A) and integrated into a label-free electrochemical aptasensor (MM_GO−AuNPs-AβO42_, Figure 10B). The thiolated aptamer specific for AβO_42_ was immobilized on the micromotor surface for selective target recognition, while AuNPs enhanced the catalytic activity by approximately two-fold. Electrochemical performance, assessed via cyclic voltammetry (CV) and electrochemical impedance spectroscopy (EIS), showed an LOD of 0.10 pg/mL and a linear range of 0.5–500 pg/mL. The aptasensor provided rapid detection within 5 min, with recovery values between 94% and 102% and relative standard deviation (RSD) below 8%. Quantification was performed directly in 5 μL of undiluted clinical samples from AD patients, demonstrating high selectivity, precision, and accuracy. Comparative analysis with a dot blot assay revealed the significantly faster performance of the micromotor-based platform: detection was completed in minutes compared to more than 14 h required for dot blot analysis of brain tissue protein extracts, CSF, and plasma containing 5.0–15.0 μg of protein. The authors highlighted the strong potential of this micromotor technology for clinical studies and point-of-care testing (POCT). However, they also noted two key challenges: adaptation of the platform for hospital implementation and the need for specialized training of healthcare personnel for its routine use.

**Figure 11:**
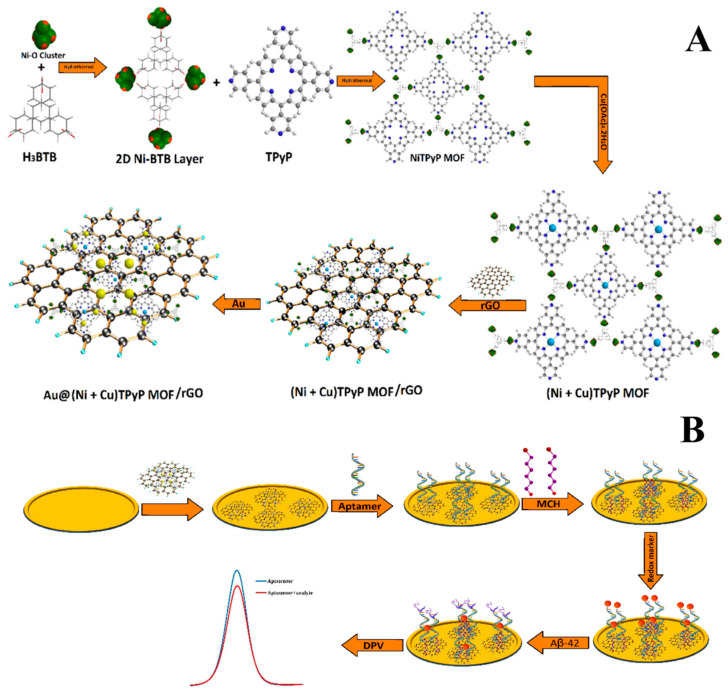
(**A**) Synthesis of Au@(Ni + Cu)TPyP MOF/rGO nanostructures. (**B**) Fabrication of the Aβ_1–42_ aptasensor (Aβ_1–42_/apt/Au@(Ni + Cu)TPyP MOF/rGO/GE). Reprinted (adapted) with permission from Vajedi et al. (2024) [124]. Copyright 2024 American Chemical Society.

An ultrasensitive electrochemical aptasensor for Aβ_42_ detection (Figure 11) was engineered by Vajedi et al. (2024) [124], combining a bimetallic nickel–cobalt–porphyrin metal–organic framework (Ni–Co–P MOF) with reduced graphene oxide (rGO) and gold nanoparticles (AuNPs) deposited on a gold electrode (GE). The integration of rGO and AuNPs improved electron transfer kinetics and enhanced conductivity, while the Ni–Co–P MOF provided a high surface area and abundant active sites for aptamer immobilization. The modified electrode exhibited approximately a sevenfold increase in electroactive surface area and significantly accelerated the Aβ_42_ redox process, as evidenced by much higher peak currents compared to the bare electrode. Analytical characterization using differential pulse voltammetry (DPV) revealed an ultralow LOD of 48.6 fg/mL and a linear range of 0.05 pg/mL to 5.00 ng/mL. The aptasensor demonstrated satisfactory stability, retaining 95.6% of its initial activity after 10 days, high reproducibility (RSD 4.3%), and negligible interference from non-target species. Practical applicability was evaluated in human blood plasma via standard addition of Aβ_42_, achieving recovery values between 95% and 104% with the DPV method. Despite its outstanding analytical performance, the complex and multi-step synthesis of the sensing platform increases production costs and limits scalability. Furthermore, the restricted assessment of long-term stability and biological interactions may hinder translation to real-world clinical applications.

The ultrasensitive detection of Aβ_1–40_ oligomers (AβO) was achieved by Fan et al. (2025) [125] using an electrochemical aptasensor based on a ternary nanocomposite composed of polypyrrole (PPy), reduced graphene oxide (rGO), and Fe_2_O_3_ nanoparticles coated on a glassy carbon electrode (GCE). This nanocomposite provided enhanced signal amplification, conductivity, and biocompatibility. Gold nanoparticles (AuNPs) were subsequently deposited to enable immobilization of an Aβ_1–40_–specific aptamer, ensuring selective target recognition. Electrochemical characterization using cyclic voltammetry (CV) and electrochemical impedance spectroscopy (EIS) confirmed the improved electroactive surface area and charge transfer properties of the modified electrode. Differential pulse voltammetry (DPV) analysis demonstrated an ultralow LOD of 40 fM within a linear range of 0.1 pM to 200 nM. The aptasensor maintained stability for up to two weeks without significant signal degradation and exhibited repeatability over five consecutive measurements, with a relative standard deviation (RSD) of 4.4%. Although the authors claimed excellent reproducibility and selectivity, no corresponding data were presented, and the distinction between repeatability and reproducibility was not clearly defined. Application of the aptasensor in artificial serum achieved recoveries between 96.3% and 105%, with RSD values below 4.5%. While the study demonstrates a promising analytical approach for AβO detection, further work is required to validate selectivity and reproducibility, assess extended stability, and perform clinical trials to confirm the platform’s utility for early AD diagnosis.

Graphene-based electrochemical biosensors for AD biomarkers have demonstrated outstanding sensitivity, with LODs ranging from 48.6 fg/mL to low picomolar levels and broad linear ranges that frequently span several orders of magnitude. The integration of graphene derivatives with metallic nanostructures (e.g., AuNPs, Pd, Co-based materials), MOFs, conducting polymers, or micromotor technology markedly improves conductivity, active surface area, and catalytic activity, thereby enhancing analytical performance. Most platforms have been validated in complex biological matrices, including human plasma, serum, CSF, and brain tissue, achieving high recoveries (>94%) and good precision (RSD typically < 5% or <8%). However, key challenges persist, particularly the complexity and time-intensiveness of fabrication protocols, limited scalability, incomplete reporting of reproducibility and selectivity, and insufficient evaluation of long-term stability. Overcoming these limitations will be essential for translating these promising sensing platforms into robust, clinically deployable diagnostic tools for early AD detection.

### 3.2. CNTs-Based Electrochemical Sensors

Similar to graphene-based nanomaterials, carbon nanotubes (CNTs) have been extensively investigated for electrochemical biosensing of Alzheimer’s disease (AD) biomarkers owing to their exceptional electrical conductivity, high surface-to-volume ratio, and capacity to promote rapid electron transfer. These intrinsic properties make CNTs highly suitable for electrode modification, contributing to enhanced sensitivity, stability, and reproducibility. Both single-walled (SWCNTs) and multi-walled CNTs (MWCNTs) have been utilized, often functionalized or combined with polymers, metal nanoparticles, or other nanostructures to improve biorecognition element immobilization and overall analytical performance. Table 3 summarizes recent CNT-based electrochemical biosensors for AD biomarker detection, detailing their detection principles, analytical performance, target analytes, sample types, and validation in real or spiked biological matrices. In addition to conventional voltammetric and impedance-based methods, recent works have also integrated CNTs into field-effect transistor (FET) architectures, an electrochemical transduction format that enables label-free and ultra-sensitive detection of multiple AD biomarkers.

**Figure 12:**
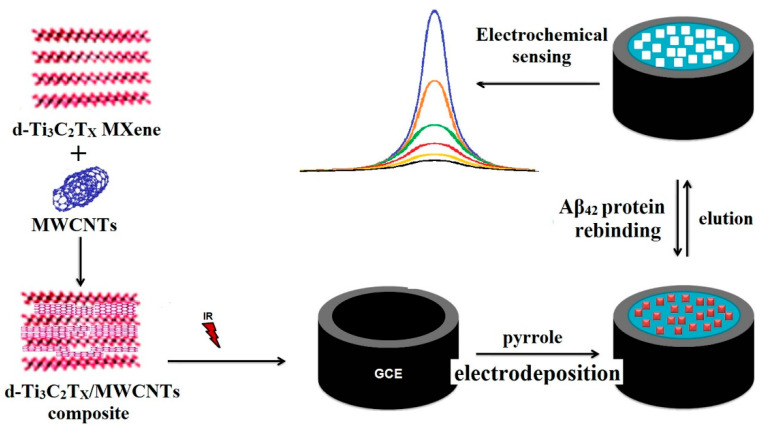
Preparation of MIP/d-Ti_3_C_2_T_x_/MWCNT-modified GCE for Aβ_1–42_ detection. Reprinted from Özcan et al. (2020) [129], Copyright (2025), with permission from Elsevier.

Özcan et al. (2020) [129] reported an ultrasensitive molecularly imprinted electrochemical biosensor for the detection of the 42-amino-acid amyloid-beta peptide (Aβ_42_), integrating delaminated titanium carbide MXene (d-Ti_3_C_2_T_x_) with multi-walled carbon nanotubes (MWCNTs) in a 3:1 mass ratio (Figure 12). The prepared d-Ti_3_C_2_T_x_ MXene and d-Ti_3_C_2_T_x_/MWCNT composite, offering high electrical conductivity and large surface area, were thoroughly characterized by UV–Vis spectroscopy, SEM, XRD, XPS, and AFM, while electrochemical behavior was evaluated by CV and EIS. The composite was then employed for the fabrication of an Aβ_42_-imprinted d-Ti_3_C_2_T_x_/MWCNTs-modified glassy carbon electrode (GCE) using polypyrrole as the imprinting matrix to create selective recognition sites. Differential pulse voltammograms (DPVs) were recorded to highlight the difference in current responses between imprinted and non-imprinted electrodes, confirming the selectivity of the molecular imprinting process. DPV was further employed for method optimization, analytical performance studies, and sample measurements. The biosensor achieved an exceptionally low LOD of 0.3 fg/mL within a narrow but ultra-sensitive linear range of 1.0–100.0 fg/mL. It exhibited remarkable robustness, with 60-day storage stability, 60-cycle repeatability, 20-device reproducibility, and 30-cycle reusability. Selectivity assays against three potential interferents, hemoglobin (HEM), heparin (HEP), and bilirubin (BIL), confirmed high specificity for Aβ_42_. Application to spiked human plasma yielded recovery values between 99.99% and 100.04%, with results in close agreement with LC-MS/MS analysis, supporting its potential for real-sample application in Alzheimer’s disease diagnostics. Despite the excellent performance, the narrow dynamic range and the multi-step imprinting process could limit direct clinical translation without further optimization. Nevertheless, the work highlights the potential of combining MXene–CNT nanostructures with molecular imprinting for next-generation, label-free Alzheimer’s disease diagnostics.

**Figure 13:**
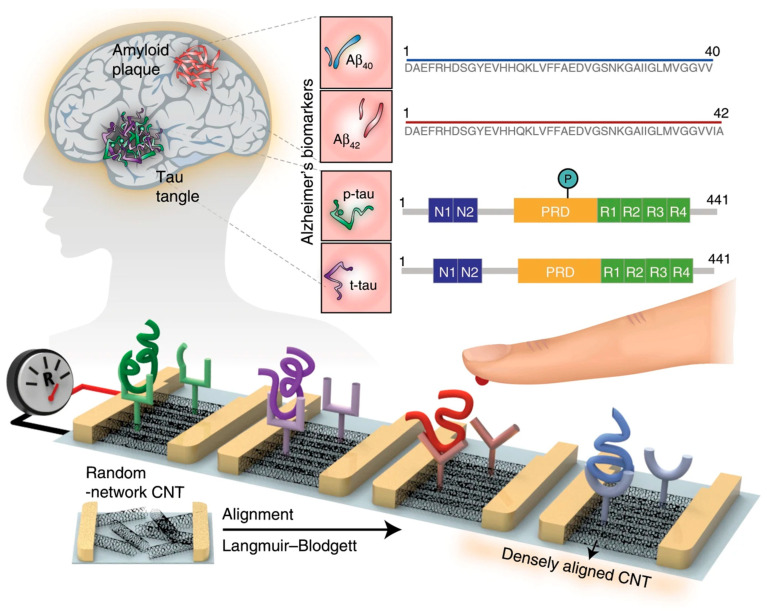
Multiplexed CNT sensor array for simultaneous detection of t-tau, p-tau181, Aβ_42_, and Aβ_40_ in plasma. Adapted from Kim et al. (2020) [6], under CC BY 4.0.

A multiplexed carbon nanotube (CNT)-based sensor array for simultaneous detection of total tau (t-tau), phosphorylated tau at threonine 181 (p-tau181), Aβ_42_, and Aβ_40_ in human plasma (Figure 13) was designed by Kim et al. (2020) [6]. Pre-fabricated CNTs were wrapped with poly[(m-phenylenevinylene)-co-(2,5-dioctoxy-p-phenylene-vinylene)] (PmPV) in dichloroethane to improve stability and ensure uniform dispersion. The CNT/PmPV suspension was sonicated for 1 h, centrifuged, filtered, and diluted before being spread onto water to form a film using the Langmuir-Blodgett method. To optimize alignment and packing density, the CNT layer was subjected to 10 compression–retraction cycles, producing a densely packed monolayer with uniform orientation. The CNT film was then transferred onto a silicon substrate and annealed to remove residual PmPV. Carboxyl functional groups were introduced via UV–ozone treatment, enabling covalent antibody immobilization through carbodiimide chemistry. Finally, metallic source and drain electrodes were deposited by e-beam evaporation to form CNT channels with controlled dimensions. Performance was evaluated using a liquid-gated transfer measurement setup with an Ag/AgCl reference electrode. Increasing concentrations of each target biomarker caused measurable increases in channel resistance, enabling a clear distinction between healthy individuals and AD patients. The array achieved LODs of 2.13 fM (Aβ_42_), 2.20 fM (Aβ_40_), 2.45 fM (t-tau), and 2.72 fM (p-tau181), with linear ranges from ~100 to 10^6^ fM. This approach demonstrated ultra-sensitive, label-free, multiplexed detection; however, validation in this study was limited to a small number of plasma samples, and no long-term stability testing was reported. Therefore, larger-scale clinical evaluation and extended stability assessment will be essential for translation to routine diagnostics.

**Figure 14:**
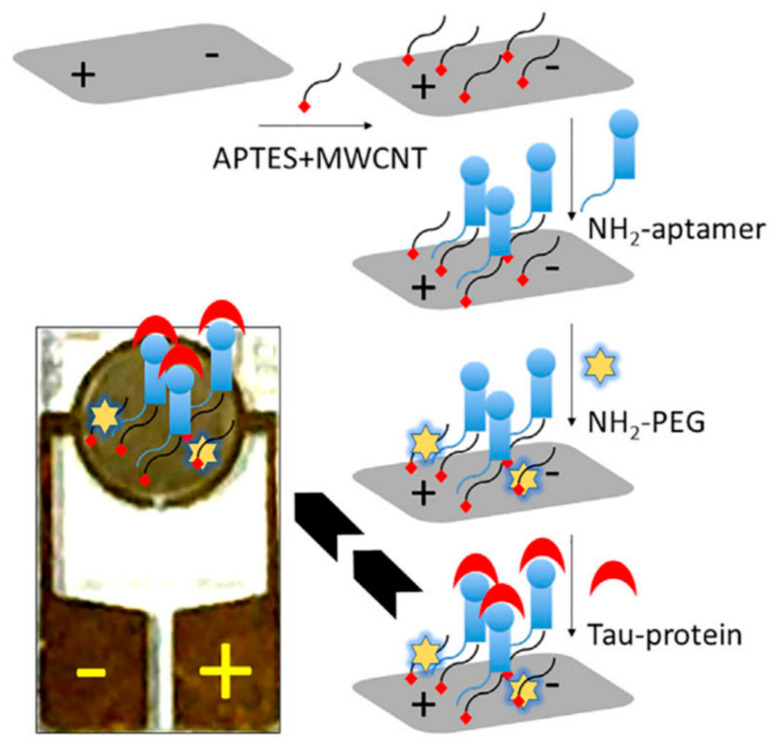
EIS aptasensor for tau protein using MWCNT-modified electrode with APTES linker, amine-aptamer immobilization, and NH_2_-PEG blocking of unmodified sites. Reproduced with permission from Yin et al. (2022) [130], Copyright [2022], with permission from Wiley.

Electrochemical impedance spectroscopy (EIS) was employed by Yin et al. (2022) [130] to construct an aptasensor for tau protein quantification, employing multi-walled carbon nanotubes (MWCNTs) functionalized with an amine-terminated tau-specific DNA aptamer (Figure 14). The high surface area and conductivity of MWCNTs provided an efficient platform for aptamer immobilization and signal transduction. In EIS, the charge transfer resistance (R_ct_) represents the resistance to electron flow between the electrode surface and a redox probe in solution and is obtained from the semicircle diameter in the Nyquist plot. Binding of target molecules to the recognition layer typically increases R_ct_ by forming a more insulating surface; however, in this system, tau protein binding caused a decrease in R_ct_, indicating that the binding event facilitated electron transfer at the electrode interface. A strong linear relationship (R^2^ = 0.9846) was observed between tau protein concentration and R_ct_ over a 1 fM–1 nM range, with an LOD of 1 fM. Specificity was verified through control experiments in which non-complementary aptamers and unrelated proteins, albumin and complement factor H (CFH), produced negligible changes in R_ct_. The sensor’s applicability was demonstrated in tau-protein-spiked human serum, where it retained high sensitivity in the complex matrix. Beyond Alzheimer’s disease research, the authors highlighted its potential for monitoring anesthesia-induced neurodegenerative conditions. Although the platform offers high sensitivity and specificity, the study’s clinical relevance remains to be established through validation in patient-derived samples.

**Figure 15:**
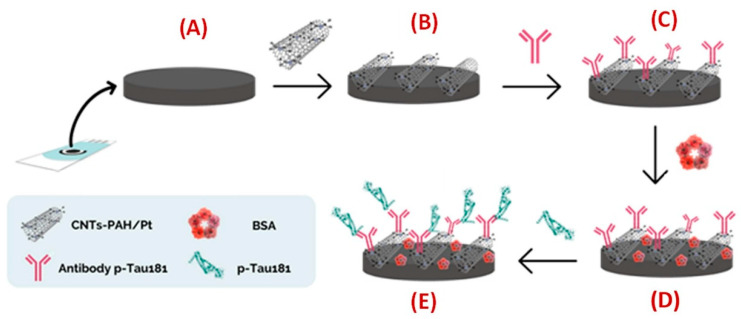
Fabrication steps of p-tau181 immunosensor using C-SPE modified with MWCNTs–PAH/Pt for antibody immobilization: (**A**) electrode pre-treatment; (**B**) deposition of the MWCNTs–PAH/Pt nanocomposite; (**C**) antibody immobilization onto the modified surface; (**D**) blocking of nonspecific adsorption sites with BSA; and (**E**) interaction of the target analyte with the sensing interface. Reprinted from Schneider et al. (2022) [131], Copyright (2022), with permission from Elsevier.

An electrochemical immunosensor for phosphorylated tau protein at threonine 181 (p-Tau181) detection was created by Schneider et al. (2022) [131] using carbon screen-printed electrodes (C-SPEs) modified with multi-walled carbon nanotubes coated with platinum nanoparticles (MWCNTs-PAH/Pt) to enable antibody immobilization (Figure 15). The nanocomposite was prepared by functionalizing MWCNTs with poly(allylamine hydrochloride) (PAH) to improve dispersion and introduce amine groups, followed by PtNP deposition. Morphology and crystallinity were characterized by transmission electron microscopy (TEM), X-ray diffraction (XRD), Raman spectroscopy, and energy-dispersive X-ray spectroscopy (EDX), while cyclic voltammetry (CV) and square-wave voltammetry (SWV) assessed electrochemical performance. The sensor achieved a limit of detection of 0.24 pg/mL and a linear range from 8.6 pg/mL to 1100 pg/mL. Selectivity was confirmed against four potential interferents, bovine serum albumin (BSA), uric acid (UA), hemoglobin (Hb), and immunoglobulin G (IgG). Real-sample applicability was evaluated in human serum spiked with p-Tau181 at 7.8 pg/mL, 15.6 pg/mL, and 1.0 ng/mL, resulting in recovery values of 95%, 87%, and 91%, respectively, using the standard addition method. The incubation time for antigen–antibody binding was set at 30 min without optimization. Although the achieved LOD is below typical physiological concentrations of p-Tau181, the authors note that several other devices in the literature have reported lower detection limits. Even though the authors affirm that the “device showed excellent reproducibility and stability”, no data on the long-term stability of the biosensor under various storage conditions and reproducibility in multiple production batches were reported. Future improvements could involve the use of more flexible, cost-effective electrodes, the incorporation of advanced nanomaterials, and multiplexed biomarker detection. While the platform shows potential for AD biomarker sensing, further optimization and clinical validation are needed for practical translation.

**Figure 16:**
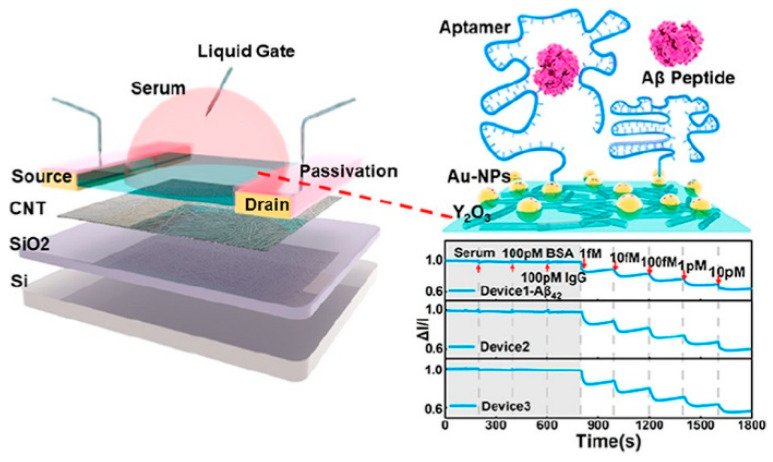
Aptamer-functionalized CNT FET biosensor for Aβ_1–42_ and Aβ_1–40_ detection in PBS and serum. Reprinted (adapted) with permission from Chen et al. (2022) [132]. Copyright 2022 American Chemical Society.

Chen et al. (2022) [132] reported the large-scale production of a single-walled carbon nanotube (SWNT)-based field-effect transistor (FET) biosensor for Aβ_42_ and Aβ_40_ detection in PBS and human serum (Figure 16). SWNTs, obtained via arc-discharge from Carbon Solution Inc., were separated using polymer-assisted sorting by Suzuki polycondensation to achieve high semiconducting purity. The sorted SWNTs were deposited onto 4-inch Si/SiO_2_ substrates to form the active transistor channels. Raman spectroscopy revealed a G/D intensity ratio of ~10:1, indicating few defects and a highly ordered structure. A thin Y_2_O_3_ gate dielectric was prepared by e-beam evaporation of Y metal followed by oxidation at 270 °C for 30 min, producing dense dielectric layers. Au nanoparticles were then deposited by e-beam evaporation onto the Y_2_O_3_ surface, allowing aptamer functionalization via Au–S bonding. The biosensor exhibited a linear response from 1 fM to 10 pM, with LODs of 45 aM for Aβ_42_ and 55 aM for Aβ_40_ in serum, the lowest reported concentrations detected in serum for these targets at the time. Sensitivity was enhanced through sequential blocking with mercaptohexanol (MCH), Tween 20, and bovine serum albumin (BSA) to minimize nonspecific adsorption. The platform showed negligible responses to albumin and human IgG, with high selectivity ratios of 730% for Aβ_40_ and 800% for Aβ_42_. This scalable FET biosensor combines exceptional sensitivity with aptamer-based specificity. Nonetheless, the work reported only short-term stability in serum (30 min) and did not include long-term performance testing or validation with large clinical cohorts. These aspects, together with optimization for extended operation in complex biological fluids, remain necessary for practical deployment.

**Figure 17:**
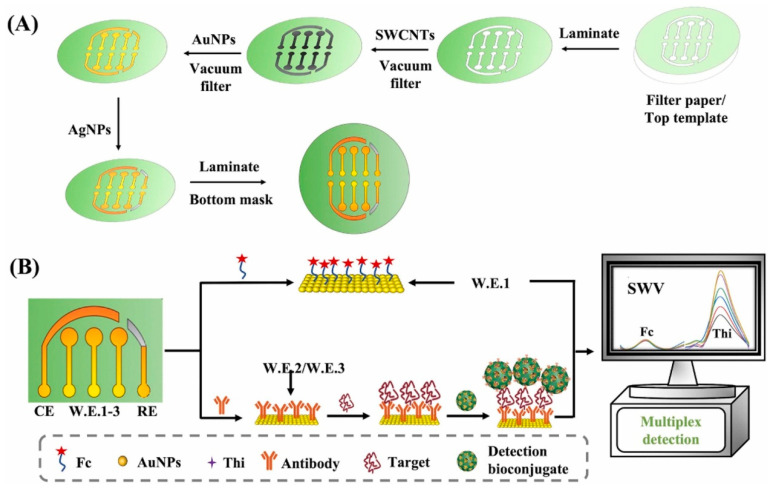
(**A**) Preparation of multiplex paper electrodes. (**B**) Immunosensor fabrication for simultaneous detection of AβO and Fetuin B. Reprinted from Gu et al. (2024) [133], Copyright (2022), with permission from Elsevier.

A multiplex paper-based electrochemical immunosensor for the simultaneous determination of amyloid beta oligomers (AβO) and Fetuin B was introduced by Gu et al. (2024) [133], who used single-walled carbon nanotubes (SWCNTs) and gold nanoparticles (AuNPs) vacuum-filtered through patterned cellulose paper to produce conductive multiplexed electrodes (Figure 17). For AβO detection, dendritic mesoporous silica nanoparticles decorated with AuNPs and loaded with thionine (dmSiO_2_–Au–Thi) served as redox labels for signal amplification, while a ferrocene (Fc)-based parallel assay was integrated for ratiometric correction in Fetuin B detection. The morphology, composition, and porosity of the nanocomposites were thoroughly characterized by SEM, TEM, FT-IR, EDS/EDX elemental mapping, XPS, XRD, UV–Vis, and N_2_ adsorption–desorption analysis, and electrochemical behavior was evaluated by cyclic voltammetry (CV) and square-wave voltammetry (SWV). Optimization studies established dmSiO_2_–Au–Thi at 1.25 mg/mL, with incubation times of 120 min (AβO–antibody binding) and 90 min (AβO–nanoconjugate binding) to maximize SWV responses. The sensor achieved LODs of 0.005 ng/mL for AβO (linear range 0.01–40 ng/mL) and 0.02 ng/mL for Fetuin B (linear range 0.05–80 ng/mL), with no cross-talk between electrodes. Selectivity tests against eleven potential interferents, namely, four metal ions (Na^+^, Fe^2+^, Ca^2+^, Cu^2+^), five amino acids (valine, cysteine, serine, glutamic acid, threonine), ascorbic acid, and dopamine, confirmed high specificity, including clear discrimination between Aβ monomers, oligomers, and fibrils. Stability studies showed 89% retention of the initial signal after 28 days. Intra-day reproducibility was evaluated using six immunosensors prepared on the same day, yielding RSDs of 2.51% for AβO and 2.96% for Fetuin B, while inter-day reproducibility over six consecutive days produced RSDs of 4.97% and 4.28%, respectively, confirming robustness and consistency. Application to hippocampus and serum samples from APP/PS1 transgenic Alzheimer’s disease mice revealed significantly higher AβO levels and decreased Fetuin B levels compared to healthy controls, supporting the link between AβO and early AD pathology, as well as inflammation-related suppression of Fetuin B. Recovery values ranged from 98.0–100.9% for AβO in hippocampus, 98.9–109.8% for AβO in serum, and 97.7–106.7% for Fetuin B in serum (all RSD < 5%). The authors noted that the multiplexing capability could be extended to other targets by replacing capture and detection antibodies, making it a flexible and cost-effective tool for rapid diagnostics. While this approach offers superior sensitivity compared to many reported electrochemical methods, some optical platforms still achieve lower absolute LODs for AβO [135]. Moreover, the relatively long incubation times (90–120 min) and multi-step synthesis could limit its direct clinical translation without further optimization.

**Figure 18:**
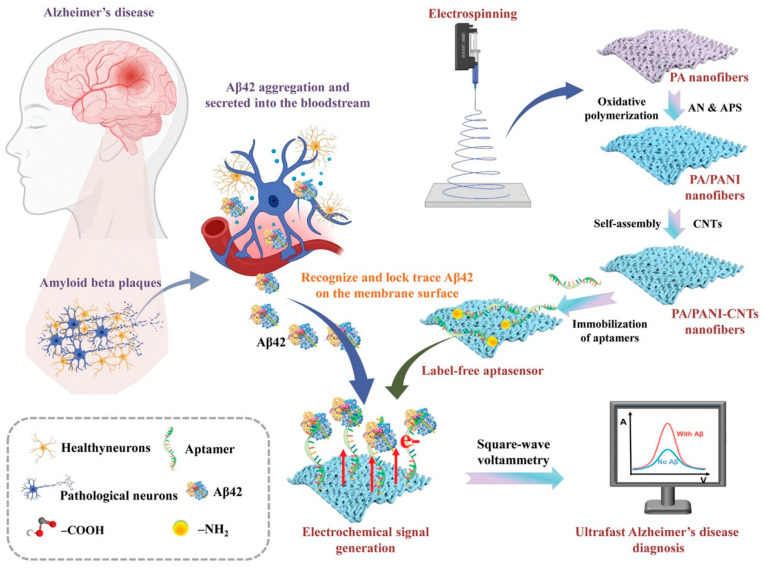
Detection mechanism of PA/PANI–CNT nanofibre-based aptasensor for rapid Aβ_1–42_ detection in human blood. Reproduced from Liu et al. (2024) [134], under CC BY-NC.

A simple, low-cost, and accessible electrochemical aptasensor for the rapid detection of Aβ_42_ in human blood (Figure 18) was proposed by Liu et al. (2024) [134]. The sensor was constructed by electrospinning polyamide (PA) nanofibers, which served as a scaffold for the deposition of polyaniline (PANI) and carbon nanotubes (CNTs) (PA/PANI–CNTs), providing abundant catalytic binding sites for the aptamer and enhancing electron transfer, as confirmed by cyclic voltammetry (CV) and electrochemical impedance spectroscopy (EIS). PA/PANI–CNTs exhibited the largest effective electrochemical surface area (0.504 cm^2^) compared to PA and PA/PANI, contributing to improved sensitivity. Biosensing performance was evaluated using square wave voltammetry (SWV), which revealed a very fast response time of 4 min, despite the requirement of a 12 h incubation step for aptamer immobilization. The aptasensor showed a 72.6% increase in current signal within 2 min, reaching a stable plateau at 4 min. The aptasensor achieved an LOD of 30 fg/mL over two linear ranges (0.1 pg/mL–500 pg/mL and 500 pg/mL–110 ng/mL). The sensor exhibited high selectivity, with minimal interference from alpha-fetoprotein (AFP), cardiac troponin I (cTnI), cancer antigen 125 (CA125), and human chorionic gonadotropin (hCG). Electrochemical stability was evidenced by only 8.35% and 7.23% decreases in oxidation and reduction peak currents after 30 CV scans, while reproducibility tests using six independently prepared aptasensors yielded an RSD of 0.96%. Long-term stability studies showed negligible degradation after 30 days. The aptasensor’s applicability was confirmed in human serum samples (*n* = 5) via SWV and the standard addition method, achieving recovery values between 99.01% and 111.50% with RSDs from 0.09% to 7.00%. Compared with other reported electrochemical Aβ_42_ sensors, which have detection times ranging from 15 to 90 min (Table S3 in [134]), this device achieved one of the fastest response times while maintaining competitive sensitivity. Despite its excellent performance, the sensor still faces two key limitations: the time-consuming aptamer immobilization process and the absence of direct clinical validation. Nevertheless, the PA/PANI–CNTs aptasensor represents a promising candidate for rapid, low-cost, and sensitive Aβ_42_ detection in early Alzheimer’s disease diagnosis.

Overall, CNT-based electrochemical biosensors for Alzheimer’s disease biomarkers (Table 3) demonstrate outstanding analytical performance, with LODs spanning from the attomolar range in FET platforms to sub-femtogram and low picogram levels in voltammetric and impedance-based sensors, and with wide linear ranges enabling both ultra-trace and broader concentration coverage. Incorporation of nanocomposites, such as MXene–CNT hybrids, polymer–CNT scaffolds, and noble metal-modified CNTs, consistently enhanced electron transfer, increased electroactive surface area, and improved signal amplification. In addition to conventional DPV, SWV, and EIS methods, recent developments have integrated CNTs into field-effect transistor (FET) architectures, enabling label-free and multiplexed detection with exceptional sensitivity. Most studies validated their platforms in spiked serum or plasma, with recoveries typically exceeding 95% and low RSD values, confirming suitability for real-sample analysis. Selectivity testing was generally robust, often including multiple physiologically relevant interferents, though the scope of interferent panels varied. Practical advantages reported include the rapid 4-min detection in Liu et al. (2024) [134], the multiplexing capability in Gu et al. (2024) [133], the simultaneous multi-biomarker detection in Kim et al. (2020) [6], and the wafer-scale fabrication approach in Chen et al. (2022) [132]. Remaining challenges include narrow dynamic ranges in some cases, multi-step fabrication processes, and limited long-term stability or batch-to-batch reproducibility data. Overall, CNT-based designs offer a promising balance between sensitivity, versatility, and clinical relevance, particularly when integrated with advanced nanostructures, scalable manufacturing, and streamlined electrode fabrication.

### 3.3. Hybrid Carbon-Based Electrochemical Sensors Involving Graphene

Carbon nanomaterial hybrids that integrate graphene derivatives with other carbon-based nanostructures, such as carbon nanotubes (CNTs) or carbon dots (CDs), have emerged as highly versatile and efficient platforms for electrochemical biosensing. By combining the unique structural and electronic features of each component, these hybrids offer synergistic enhancements in electroactive surface area, charge-transfer kinetics, dispersion stability, and mechanical robustness. For instance, CNTs contribute a one-dimensional high aspect ratio and outstanding electrical conductivity, while CDs provide abundant surface functional groups, tunable photoluminescence, and nanoscale dimensions that facilitate homogeneous dispersion and electron transfer. In both cases, the two-dimensional graphene framework delivers a high surface-to-volume ratio, excellent electron mobility, and a conductive scaffold for immobilizing recognition elements.

Functionalization of graphene-based hybrids with polymers, small molecules, or nanoparticles further tailors their physicochemical and biocompatibility profiles, enabling the design of robust biosensor architectures. In Alzheimer’s disease (AD) diagnostics, such hybrids have been exploited to improve sensitivity, selectivity, and stability in complex matrices such as serum. Reported approaches have incorporated biorecognition strategies including aptamers, antibodies, and molecularly imprinted polymers (MIPs), often in combination with signal amplification schemes using metallic nanostructures or redox mediators. Table 4 summarizes representative electrochemical biosensors based on graphene–carbon nanomaterial hybrids for AD biomarker detection, highlighting their detection principles, analytical performance, target analytes, sample types, and validation approaches.

Li et al. (2020) [136] developed a multi-amplified electrochemical biosensor for tau-441 protein detection by modifying a gold electrode (GE) with a nanocomposite film of multi-walled carbon nanotubes (MWCNTs), reduced graphene oxide (rGO) and chitosan (CS). The MWCNTs-rGO-CS composite was prepared by ultrasonic dispersion of MWCNTs, rGO, and CS in 2% acetic acid, exploiting the synergy between MWCNTs and rGO to improve conductivity, dispersibility, and active surface area. CS provided excellent film-forming ability and biocompatibility, serving as a stable matrix for antibody immobilization. The film was drop-cast onto a pretreated GE and crosslinked with glutaric dialdehyde (GLA) to covalently attach the specific anti–tau-441 antibody. For further signal amplification, AuNPs synthesized by sodium borohydride reduction and functionalized with cysteamine were conjugated to tau-441 protein, enabling Au–S bonding and enhanced electron transfer blocking upon antigen–antibody recognition. Detection was performed by differential pulse voltammetry (DPV) using [Fe(CN)_6_]^3−^/^4−^ as the redox probe. The sensor achieved an LOD of 0.46 fM over a range of 0.5–80 fM, with high reproducibility (RSD = 4.74%) and good stability (92.86% activity retained after 11 days). Selectivity tests showed minimal signal variation (<5% interference) in the presence of glucose, ascorbic acid, L-cysteine, α-synuclein, and human serum albumin. Recovery values in spiked serum ranged from 90.67% to 102.33% (RSD < 5%). Real sample validation with human serum from 14 healthy individuals, 14 mild cognitive impairment patients, and 14 dementia patients revealed a significant correlation between tau-441 levels and cognitive impairment severity, demonstrating the potential of this non-invasive platform for early dementia diagnosis.

**Figure 19:**
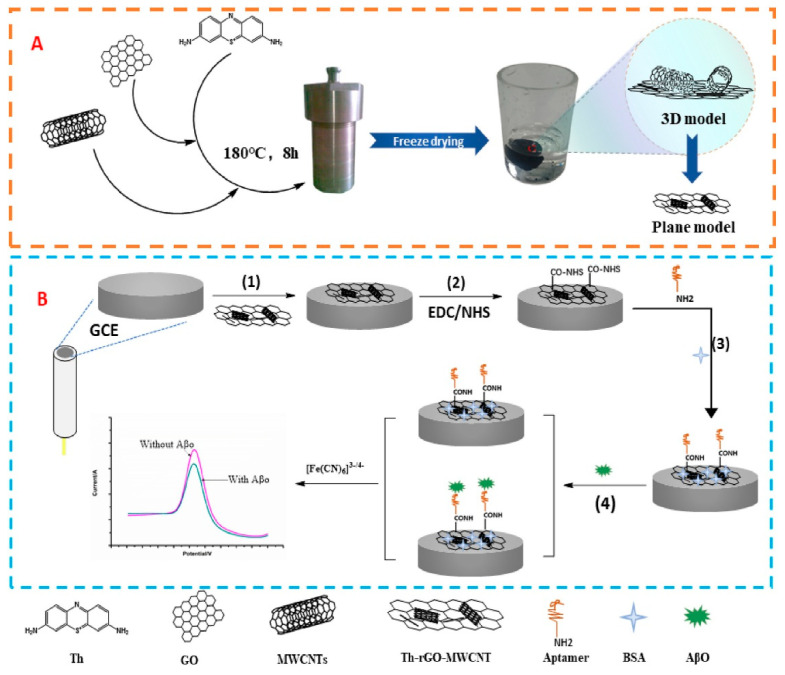
(**A**) Synthesis of Th–rGO–MWCNT nanocomposite. (**B**) Aptasensor assembly for AβO detection with BSA blocking of non-specific sites: (1) deposition of the Th–rGO–MWCNT nanocomposite as a thin conductive film to enhance electron transfer; (2) activation of surface carboxyl groups with EDC/NHS to enable covalent coupling; (3) immobilization of the aptamer followed by BSA treatment to block nonspecific adsorption; and (4) binding of Aβ oligomers to the surface-anchored aptamer during the sensing event. Reprinted from Tao et al. (2021) [137], Copyright (2021), with permission from Elsevier.

For amyloid-beta oligomer (AβO) detection, Tao et al. (2021) [137] designed an electrochemical aptasensor based on thionine (Th)-functionalized three-dimensional carbon nanomaterials combining rGO and MWCNTs (Figure 19). Thionine, a positively charged planar aromatic molecule, was incorporated via π–π conjugation with rGO and MWCNTs, enhancing structural stability, electron transfer, and capacitive properties. The Th-rGO-MWCNTs nanocomposite was synthesized through a one-step hydrothermal process with urea as the reducing agent, drop-cast onto a pretreated glassy carbon electrode (GCE), and activated with EDC/NHS (1-ethyl-3-(3-dimethylaminopropyl)carbodiimide/N-hydroxysuccinimide) chemistry for covalent attachment of an amino-modified DNA aptamer specific to AβO. Bovine serum albumin (BSA) was used to block non-specific binding sites. Differential pulse voltammetry (DPV) using [Fe(CN)_6_]^3−^/^4−^ as a redox probe revealed decreasing peak currents with increasing AβO concentration, attributed to the formation of an aptamer–AβO complex hindering electron transfer. The aptasensor exhibited an LOD of 10 fM and a linear range from 0.0443 pM to 443.00 pM under optimized conditions (pH 7.4, aptamer concentration 5 μM, 20 min incubation). Selectivity tests against Aβ monomers, Aβ fibrils, α-synuclein oligomers, and tau protein (all at 4.43 pM) showed a significant current change only for AβO, confirming high specificity. The device showed excellent reproducibility (RSD < 2%) and stability, retaining 90% of its initial signal after 16 days at 4 °C. Application to diluted human serum spiked with AβO (0.0443 pM–44.30 pM) yielded a correlation coefficient of 0.991 between measured and expected values, with recoveries between 99.71% and 103.84% (RSD < 1%). Real sample testing involved serum from 20 volunteers: 10 with Mini-Mental State Examination (MMSE) scores > 27 and 10 with scores between 10 and 20. The sensor detected markedly higher AβO levels in the latter group (0.2139 ± 0.0015 pM) compared to the first (<10 fM), highlighting its potential for early Alzheimer’s disease diagnosis. Reported limitations include the small number of clinical samples and the absence of extended stability testing in real serum.

For Aβ oligomer (AβO) detection, Negahdary et al. (2023) [138] developed an electrochemical aptasensor by modifying a glassy carbon electrode (GCE) with electrodeposited jagged gold (JG) nanostructures and a graphene oxide-carboxylated multi-walled carbon nanotube (GO-c-MWCNTs) nanocomposite. The JG nanostructure, formed by chronoamperometry (0 V, 300 s), offered high surface roughness and conductivity, facilitating electron transfer. The GO-c-MWCNTs layer combined the large surface area of GO with the excellent conductivity of MWCNTs, while carboxylic functional groups improved aptamer immobilization through covalent EDC/NHS coupling, ensuring strong and oriented DNA strand attachment. This hybrid structure increased electroactive surface area, improved electron transfer kinetics, and enhanced structural stability by preventing CNT aggregation. Amino-terminated DNA aptamers specific for AβO were immobilized onto the nanocomposite surface, with non-specific sites blocked by BSA. Electrochemical characterization (CV, EIS, DPV) confirmed a significant decrease in charge-transfer resistance and an increase in current density after modification. Under optimized binding conditions (20 min incubation), the aptasensor achieved an LOD of 0.088 pg/mL and a linear range of 0.1 pg/mL–1 ng/mL. It exhibited high stability (signal retention > 98% after 11 days, RSD = 1.22%), reproducibility (RSD < 2% over five fabrications), and reversibility (three regeneration cycles using hot-water treatment). Selectivity tests against Aβ monomers (ABMs) and fibrils (ABFs) at 0.1 and 1 ng/mL, including mixtures with AβO, showed negligible interference. Real-sample analysis in serum from 10 volunteers yielded recoveries of 93–110%, demonstrating accurate detection in complex biological matrices. The authors highlighted the synergistic benefits of the JG/GO-c-MWCNTs interface for signal amplification and aptamer immobilization, and suggested future work should focus on more flexible, low-cost electrode platforms, alternative nanomaterial assemblies, and multiplexed AD biomarker detection.

**Figure 20:**
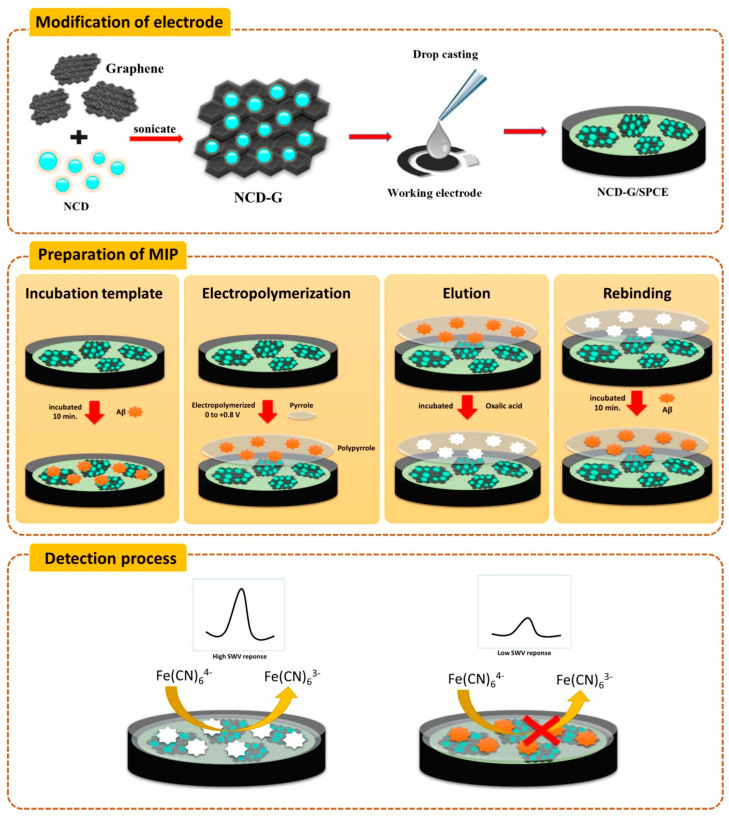
Preparation of NCD–G/SPCE, MIP formation, and detection process for Aβ_1–42_. Reprinted from Pakapongpan et al. (2024) [139], Copyright (2024), with permission from Elsevier.

For Aβ_42_ detection, Pakapongpan et al. (2024) [139] reported a disposable electrochemical sensor based on a molecularly imprinted polymer (MIP) formed on a nitrogen-doped carbon dot–graphene (NCD–G) nanohybrid platform (Figure 20). The NCD–G was synthesized via ultrasonication, exploiting π–π interactions between the 0D NCDs and 2D graphene to prevent sheet aggregation, increase solubility, and enhance electrical conductivity. This nanohybrid was drop-cast onto a screen-printed carbon electrode (SPCE) and electropolymerized with polypyrrole in the presence of the Aβ_42_ template, producing MIP films with specific recognition sites. Surface morphology and composition were characterized by SEM, TEM, UV–Vis, and XPS, while electrochemical behavior was evaluated by CV and SWV. Key parameters, including NCD–G concentration, monomer-to-template ratio, polymerization cycles, and elution/rebinding times, were optimized. The resulting MIP/NCD–G/SPCE exhibited a linear range of 5–70 pg/mL and an LOD of 1 pg/mL, with high selectivity against three structurally related proteins (BNP, IgG, and HSA) and excellent reproducibility (RSD 2.08%, *n* = 15). Validation in artificial human serum (*n* = 3) using the standard addition method yielded recoveries of 92.31–119.25% with RSD ≤ 5.44%. The single-use, low-cost platform demonstrates the potential of NCD–G–MIP integration for on-site point-of-care Alzheimer’s diagnostics; however, further evaluation with clinical samples, comparison with standard assays such as ELISA, and investigation of possible cross-reactivity with peptides such as Aβ_40_ are required for clinical translation.

The graphene-based hybrid biosensors reviewed in this section exhibit remarkable analytical sensitivity, with limits of detection spanning from sub-femtomolar (Li et al., 2020 [136]) to low pg/mL levels (Negahdary et al., 2023 [138]; Pakapongpan et al., 2024 [139]), and linear ranges suitable for clinically relevant biomarker quantification. Differential pulse voltammetry remains the predominant detection method, although cyclic voltammetry and square wave voltammetry have also been employed, typically using ferricyanide or intrinsic redox mediators for signal transduction. The targeted biomarkers included tau-441 protein, Aβ oligomers, and Aβ_42_, using aptamer-, antibody-, or molecularly imprinted polymer-based recognition elements to ensure high selectivity even in the presence of structurally related molecules. All reviewed sensors achieved high accuracy and reproducibility, with most validated in human serum, though validation in artificial serum was reported for the CD/graphene hybrid. Common strengths include the synergistic enhancement of conductivity and electroactive surface area from combining graphene with other carbon nanomaterials, improved dispersion stability, and compatibility with signal amplification or multiplexing strategies. Frequent limitations are the small number of clinical samples tested, the absence of long-term stability studies in complex biological fluids, and the need to develop scalable, cost-effective fabrication methods for integration into routine diagnostic applications.

### 3.4. Carbon Nitride (g-C_3_N_4_)-Based Electrochemical Sensors

Graphitic carbon nitride (g-C_3_N_4_) is a two-dimensional, polymeric semiconductor composed mainly of carbon and nitrogen atoms in tri-s-triazine units. Its extended π-conjugation, tunable band gap, high surface area, and chemical stability make it attractive for electrochemical and photoelectrochemical biosensing applications. As a metal-free material, g-C_3_N_4_ can be synthesized from inexpensive precursors (e.g., urea, melamine, dicyandiamide) via thermal polymerization, offering low toxicity and environmental compatibility. In biosensors, it serves as both a conductive substrate and a luminophore, enhancing electron transfer kinetics and providing abundant active sites for immobilization of biorecognition elements.

To further improve sensitivity, g-C_3_N_4_ can be integrated into composite architectures with metals, metal oxides, or other nanomaterials, enabling synergistic effects such as improved charge separation, catalytic activity, and signal amplification. This strategy has been applied to Alzheimer’s disease (AD) biomarker detection, where g-C_3_N_4_-based materials have been used to construct ultrasensitive aptasensors for amyloid-β species. Table 5 summarizes the reported g-C_3_N_4_-based electrochemical and photoelectrochemical biosensors for AD biomarker detection, highlighting their detection method, sensing platform, analytical performance, target/sample type, and validation with real samples.

**Figure 21:**
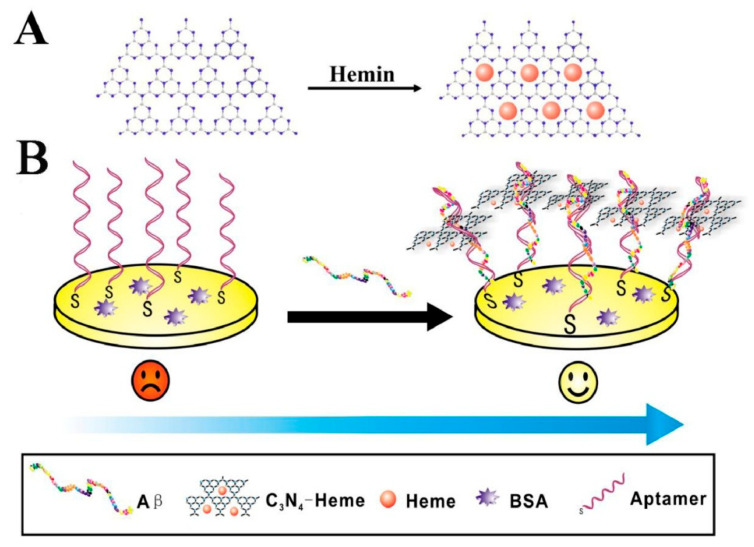
ECL-based detection of Aβ using g-C_3_N_4_–heme nanocomposite. (**A**) Formation of the g-C_3_N_4_–heme nanocomposite through coordination of hemin with g-C_3_N_4_ nanosheets. (**B**) Stepwise assembly of the aptasensor on a gold electrode: aptamer immobilization and BSA blocking yield a weak ECL signal, whereas Aβ binding recruits g-C_3_N_4_–heme via heme–Aβ interaction, producing an enhanced cathodic response. Reprinted from Zhang et al. (2020) [140], Copyright (2020), with permission from Elsevier.

A dual-enhanced electrochemiluminescence (ECL) biosensor for amyloid-β (Aβ) detection was created by Zhang et al. (2020) [140] by exploiting a nanocomposite of two-dimensional graphite-like carbon nitride (g-C_3_N_4_) and heme, assembled via π–π interactions. In this design (Figure 21), g-C_3_N_4_ acted as a metal-free ECL luminophore with high chemical stability and surface area, while heme contributed peroxidase-like catalytic activity due to its biological affinity for Aβ. A thiol-functionalized DNA aptamer specific to Aβ_40_ was immobilized on a gold electrode (GE) and subsequently incubated with the target peptide and the g-C_3_N_4_–heme nanocomposite. Upon aptamer–Aβ binding, the heme moiety catalyzed the in situ generation of hydrogen peroxide (H_2_O_2_) from dissolved oxygen via the Aβ–heme interaction. Together with potassium persulfate (K_2_S_2_O_8_) present in the electrolyte, this in situ-produced H_2_O_2_ acted as a dual co-reactant system, significantly enhancing the cathodic ECL emission of g-C_3_N_4_ at ~460 nm without the need for externally added H_2_O_2_. Material characterization by TEM, UV–vis, FT-IR, and XPS confirmed the successful integration of heme into g-C_3_N_4_ nanosheets, preserving the bulk g-C_3_N_4_ structure while incorporating both Fe^2+^ and Fe^3+^ oxidation states. Electrochemical impedance spectroscopy (EIS) showed reduced charge-transfer resistance after g-C_3_N_4_–heme–Aβ complex formation, indicating enhanced electron transfer at the interface. Control experiments with methylene blue (O_2_ reduction inhibitor) and N_2_-saturated conditions confirmed the essential role of dissolved oxygen in H_2_O_2_ generation and signal amplification. A xylenol orange assay further validated in situ H_2_O_2_ production. Optimization studies established a 1:1 g-C_3_N_4_:heme mass ratio, pH 7.4, a 12 h incubation time, and a 100 mV s^−1^ scan rate as optimal conditions. Under these parameters, the aptasensor achieved a wide linear range (10 fM–0.1 μM) and an ultralow limit of detection (LOD) of 3.25 fM. The sensor demonstrated adequate operational stability (no significant ECL changes after 200 s of cyclic potential scanning), reproducibility (RSD 4.65% for *n* = 5 electrodes), and selectivity towards monomeric Aβ over oligomers, fibrils, and unrelated proteins (BSA, CEA, thrombin). Notably, TEM revealed that heme promoted Aβ aggregation into denser oligomers, which were not effectively recognized by the aptamer, explaining the lower ECL response for aggregated forms. The assay was validated in spiked human serum (*n* = 3) using the standard addition method, yielding recoveries of 95.3%, 97.7%, and 104.1% (no RSD reported). However, the study did not include long-term stability assessment or cross-reactivity testing against other amyloid proteins. While clinical validation and usability improvements are still required for real-world deployment, this platform demonstrates the potential of g-C_3_N_4_–heme-based ECL sensing for early Alzheimer’s disease diagnosis or for monitoring therapeutic interventions, combining high sensitivity with a straightforward label-free detection format.

**Figure 22:**
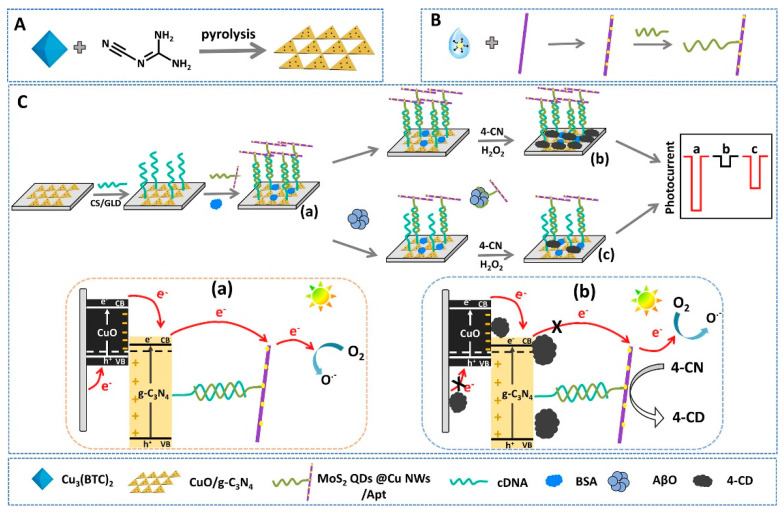
(**A**) Synthesis of CuO/g-C_3_N_4_. (**B**) Preparation of MoS_2_ QDs@Cu/Apt conjugate. (**C**) Fabrication and sensing mechanism of PEC aptasensor for AβO. Each of (a–c) includes a schematic illustration and the corresponding photocurrent response: (a) hybridization of surface-anchored cDNA with the aptamer–nanocomposite produces the initial photocurrent (“on” state); (b) in the absence of AβO, nanozyme-catalyzed oxidation of 4-CN forms insulating 4-CD, suppressing the photocurrent (“off” state); (c) upon AβO binding, the aptamer–nanocomposite dissociates, reducing 4-CD formation and restoring the photocurrent (“on” state). Reprinted from Zhang et al. (2021) [141], Copyright (2021), with permission from Elsevier.

For ultrasensitive amyloid-β oligomer (AβO) detection, Zhang et al. (2021) [141] developed a cathodic photoelectrochemical (PEC) aptasensor integrating a CuO/g-C_3_N_4_ p–n heterojunction photocathode with MoS_2_ quantum dots–decorated copper nanowires (MoS_2_ QDs@Cu NWs) as a multifunctional signal amplifier (Figure 22). The CuO/g-C_3_N_4_ photocathode was synthesized via in situ pyrolysis of a copper-based metal–organic framework (Cu-MOF) and dicyandiamide, producing a heterojunction with enhanced charge separation and visible-light absorption. MoS_2_ QDs@Cu NWs, prepared by electrostatic self-assembly, served a dual role: improving the PEC signal and functioning as a nanozyme to catalyze the oxidation of 4-chloro-1-naphthol (4-CN) in the presence of H_2_O_2_, generating an insulating precipitate that further modulated the photocurrent. The sensing interface was constructed by immobilizing a partly complementary DNA strand (cDNA) on the CuO/g-C_3_N_4_ photocathode. Hybridization with an aptamer-labelled MoS_2_ QDs@Cu NWs conjugate completed the initial “signal-on” configuration. In the presence of target AβO, the aptamer preferentially bound the oligomers, dissociating from the cDNA and releasing the MoS_2_ QDs@Cu NWs from the electrode surface, resulting in a measurable photocurrent change. This “on–off” PEC strategy ensured low background signal and high specificity. Structural and compositional characterization (SEM, TEM, XRD, XPS, zeta potential) confirmed the successful formation of the CuO/g-C_3_N_4_ heterojunction and MoS_2_ QDs@Cu NWs composite. EIS studies demonstrated efficient charge transfer across the heterojunction, while peroxidase-like activity assays verified the catalytic capability of the MoS_2_ QDs@Cu NWs. Optimization experiments identified 0.60% CuO loading, –0.2 V applied potential, 4 μM cDNA concentration, 45 min aptamer–nanocomposite incubation, 20 min catalytic precipitation time, and 1 h AβO incubation as optimal conditions. Under these conditions, the aptasensor achieved an exceptionally wide linear range (10 fM–0.5 μM) with an ultralow LOD of 5.79 fM, calculated from a signal-to-noise ratio of 3. The platform demonstrated high selectivity for AβO over five potential interfering species (TNF-α, BSA, lysozyme, insulin, horseradish peroxidase), adequate short-term stability (101.13% of initial signal after 10 light on/off cycles), and long-term stability (91.69% of initial signal after 2 weeks at 4 °C). Reproducibility was confirmed with an RSD of 3.35% for six independently prepared sensors. Practical applicability was validated in two serum samples, with recoveries ranging from 98.20% to 103.12% and RSDs of 3.31–3.49%. Despite its excellent analytical performance, the authors noted that translation to clinical use would benefit from simplifying the multi-step assembly and validation with patient-derived samples in complex matrices. This work highlights the synergistic use of g-C_3_N_4_-based heterojunctions and multifunctional nanozymes for ultrasensitive PEC biosensing, establishing a promising foundation for next-generation Alzheimer’s diagnostics.

A photoelectrochemical (PEC) aptasensor for amyloid-β40 (Aβ_40_) was reported by Li et al. (2025) [90], employing a TiO_2_/Au-g-C_3_N_4_ heterojunction to enhance interfacial charge transfer efficiency and provide abundant Au–S binding sites for aptamer immobilization and enhancing their capture efficiency. The sensing interface was constructed by electrophoretic deposition of TiO_2_ nanosheets onto the fluorine-doped tin oxide (FTO) surface, followed by annealing at 400 °C for 2 h, coating with Au-decorated g-C_3_N_4_, and a second annealing step at 250 °C for 30 min. A thiolated Aβ_40_ aptamer was assembled onto the gold surface and passivated with 6-mercapto-1-hexanol (MCH). Upon Aβ_40_ binding, the aptamer–target complex altered the interfacial charge environment, resulting in a concentration-dependent increase in photocurrent (“signal-on” PEC response). Material and interfacial characterization was carried out by SEM, HRTEM with EDS mapping, UV–visible diffuse reflectance (UV–Vis DRS), XRD, XPS, EIS, chronocoulometry (CC), confocal laser scanning microscopy (CLSM), and photocurrent-time (i–t) measurements under potentiostatic polarization. The TiO_2_/Au-g-C_3_N_4_ heterojunction was reported to facilitate charge separation under illumination, while Au nanoparticles contributed to improved conductivity and aptamer immobilization, resulting in strong photocurrent amplification. The aptasensor achieved an ultralow LOD of 0.33 fg/mL and a linear range from 10^−15^ to 10^−11^ g/mL in PBS, with comparable performance in artificial saliva, cerebrospinal fluid (CSF), and plasma. Selectivity was confirmed against tau protein, Aβ_42_, ascorbic acid, glucose, urea, and chitosan at a 1000-fold excess. Stability assessment showed only 6.9% signal loss after nine days at room temperature, and reproducibility across six independently fabricated electrodes gave an RSD of 2.69%. Importantly, clinical validation was attempted using diluted CSF (*n* = 3) and plasma (*n* = 6) from both AD and non-AD subjects, with results consistent with single-molecule array (SiMoA) measurements and per-sample RSDs below 5.9%. Despite these promising attributes, the study was limited by the small number of clinical samples (CSF *n* = 3; plasma *n* = 6), the requirement for a 1000-fold dilution of biological matrices, and the absence of long-term stability data beyond the 9-day assay performed. Nevertheless, the straightforward electrode fabrication and robust anti-interference capability highlight the potential of TiO_2_/Au-g-C_3_N_4_ composites for PEC biosensing of Aβ_40_ in early Alzheimer’s disease diagnostics.

The three studies reviewed in this section illustrate the versatility of g-C_3_N_4_ in Alzheimer’s disease biosensing, serving either as a luminophore in electrochemiluminescence (ECL) platforms or as a semiconductor scaffold in photoelectrochemical (PEC) architectures. Coupling g-C_3_N_4_ with catalytically active nanomaterials, such as heme or MoS_2_ quantum dots-decorated copper nanowires (MoS_2_ QDs@Cu NWs), enabled dual-signal amplification strategies that achieved femtomolar LODs for Aβ_40_ monomers and Aβ oligomers in spiked serum. More recently, the integration of g-C_3_N_4_ with TiO_2_ and Au nanoparticles produced a heterojunction photoanode capable of detecting Aβ_40_ down to the femtogram per milliliter level, with validation in diluted CSF and plasma samples from AD and non-AD subjects. Collectively, these works highlight how g-C_3_N_4_-based composites support ultrasensitive detection of different amyloid-β species across both ECL and PEC formats, achieving high selectivity, reproducibility, and short-term stability. Nonetheless, clinical translation will require simplification of multi-step fabrication, evaluation of performance in minimally processed biological fluids, and validation with larger patient cohorts over extended storage periods. Nonetheless, clinical translation will require simplification of multi-step fabrication, assessment of robustness in complex biological fluids, and validation with patient-derived samples.

### 3.5. Other Carbon-Based Electrochemical Sensors

Beyond graphene, carbon nanotubes, and graphitic carbon nitride, other carbon-based nanomaterials such as carbon black, carbon fiber paper, and nanoporous carbons have also been explored for electrochemical biosensing of Alzheimer’s disease (AD) biomarkers. These materials can offer distinct advantages, including low cost, biodegradability, large electroactive surface areas, and inherent antifouling properties, while remaining compatible with versatile electrode fabrication approaches. Often integrated with aptamer recognition elements, molecularly imprinted polymers (MIPs), or nanoparticle modifiers, these platforms have achieved impressive detection limits, broad dynamic ranges, and robust selectivity in complex matrices. Table 6 summarizes representative examples of such carbon-based electrochemical biosensors, outlining their detection strategies, analytical performance, targets, sample types, and validation approaches.

**Figure 23:**
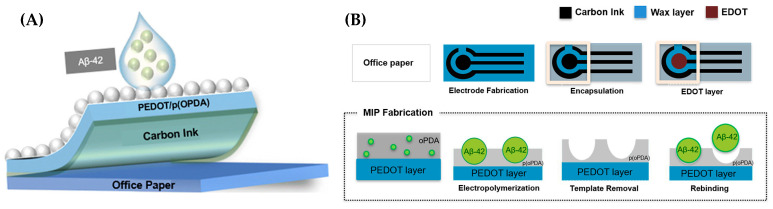
(**A**) Layer structure of paper-based electrode with PEDOT, carbon ink, MIP (o-phenylenediamine), and Aβ_1–42_. (**B**) Workflow for MIP fabrication on carbon-ink paper electrodes for Aβ_1–42_ detection. Adapted from Pereira et al. (2020) [142], *ACS Omega* **2020**, *5*, 12057–12066 (https://doi.org/10.1021/acsomega.0c00062). © 2020 American Chemical Society.

Pereira et al. (2020) [142] developed a low-cost, biodegradable, paper-based electrochemical aptasensor for the detection of amyloid-β_42_ (Aβ_42_) in Cormay serum solution, integrating a molecularly imprinted polymer (MIP) for enhanced specificity (Figure 23). The sensing platform was fabricated on office paper coated with conductive poly(3,4-ethylenedioxythiophene) (PEDOT), prepared by in situ electropolymerization of 3,4-ethylenedioxythiophene (EDOT), and functionalized with a carbon ink electrode. The MIP was formed by electropolymerizing *o*-phenylenediamine in the presence of the Aβ_42_ template, creating selective binding sites complementary to the target (Figure 23B). Electrochemical characterization by cyclic voltammetry (CV), square wave voltammetry (SWV), and electrochemical impedance spectroscopy (EIS) demonstrated that the MIP-modified paper electrode exhibited a marked response to Aβ_42_, with an LOD of 0.067 ng/mL and a linear range from 0.1 ng/mL to 1 μg/mL in serum. Surface and chemical analyses by Raman spectroscopy, scanning electron microscopy (SEM), and atomic force microscopy (AFM) confirmed the successful synthesis and deposition of the PEDOT and MIP layers. The sensor achieved a 20 min detection time and demonstrated good selectivity, producing a 27% relative signal change for the target Aβ_42_, while the potential interferents BSA (4 mg/mL), glucose (0.7 mg/mL), and creatinine (1 μg/mL) yielded only ~3%, ~1%, and ~0% signal changes, respectively. Although the authors stated that the aptasensor possessed high chemical stability, reproducibility, and repeatability (<10% RSD), no detailed data were reported to support these claims. The platform was also described as eco-friendly and extremely inexpensive, with an estimated production cost of approximately €0.03 per sensor due to the use of paper substrates and inexpensive conductive inks. Importantly, given that healthy individuals typically exhibit Aβ_42_ concentrations around 23.3 pg/mL, the biosensor’s low LOD allows detection within physiologically relevant levels, even in complex matrices such as serum. Although the specific composition of the carbon ink was not disclosed, such formulations often contain carbon black as the conductive filler [145], suggesting that this work may also be regarded as an application of carbon black-based electrodes. By combining the eco-friendly and disposable nature of paper-based substrates with the specificity of MIP recognition, this aptasensor represents a promising approach for point-of-care (POC) Alzheimer’s diagnostics. Nevertheless, long-term stability assessment, multiplexing capability, clinical validation, and integration with portable readout electronics remain essential steps for real-world translation.

**Figure 24:**
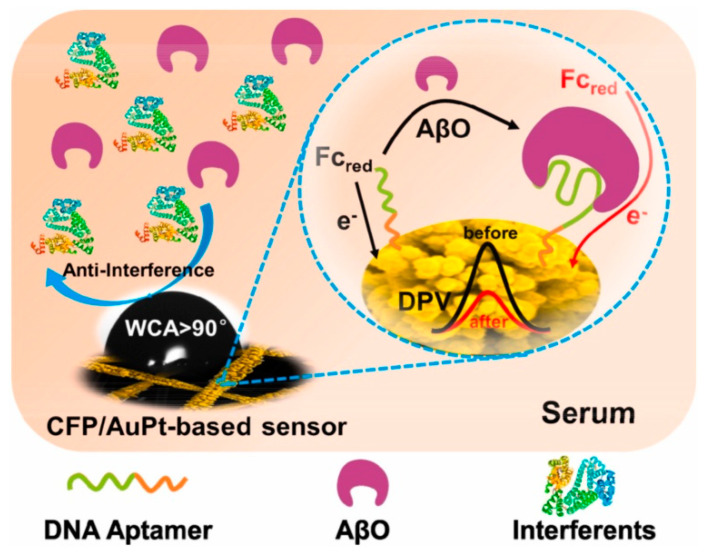
Electrochemical aptasensor for AβO detection based on superhydrophobic CFP/AuPt nanocomposite. Reprinted from Liu et al. (2021) [143], Copyright (2021), with permission from Elsevier.

Integration of structural hydrophobicity with catalytic nanostructures was the focus in Liu et al. (2021) [143], where superhydrophobic carbon fiber paper (CFP) was combined with electrodeposited AuPt alloy nanoparticles to form CFP/AuPt nanocomposites for Aβ oligomer (AβO) detection (Figure 24). This dual-functional surface combined a large electroactive area, which increased the number of active sites and facilitated electron transfer, with enhanced resistance to nonspecific adsorption, thereby minimizing signal loss in complex biological matrices. Thiolated DNA aptamers targeting AβO were covalently anchored to the CFP/AuPt surface via self-assembly, ensuring stable and oriented probe immobilization, while ferrocene acted as a well-defined redox mediator for sensitive DPV signal transduction. The electrode architecture and operational parameters, including HAuCl_4_/H_2_PtCl_4_ ratio, deposition voltage and time, and aptamer concentration, were optimized by CV, while surface morphology was characterized by SEM and EDX mapping. Under optimized conditions, DPV measurements revealed a wide linear range of 0.5–10,000 pg/mL and an exceptionally low LOD of 0.16 pg/mL (LOQ 0.48 pg/mL). The aptasensor exhibited high selectivity against possible interferents, including Aβ_1–40_ and Aβ_1–42_ monomers, tau protein, and neurofilament light protein (NFL). In spiked human serum, the device maintained 90% of its initial current after 168 h incubation, achieving recoveries of 92.5–109% with RSD < 10%, and showed lower LOD and LOQ values compared to ELISA. Despite the impressive antifouling capability and analytical performance, reproducibility and long-term shelf-life assessments were not reported, and validation with clinical AD patient samples remains necessary for future application.

**Figure 25:**
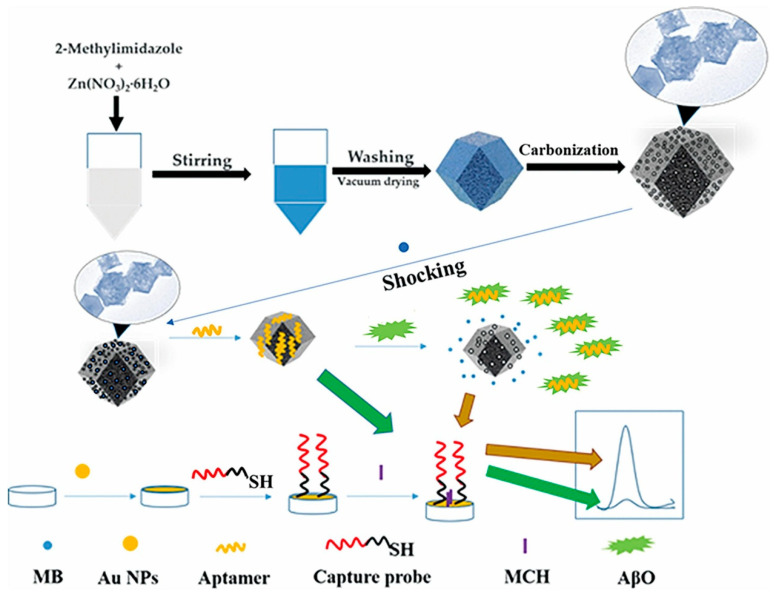
Synthesis of nanoporous carbon and AβO detection using MB-tagged aptasensor on AuNP-modified GCE. Reprinted from Ren et al. (2023) [144], Copyright (2023), with permission from Elsevier.

For Aβ oligomer (AβO) detection, Ren et al. (2023) [144] assembled a highly sensitive, specific, and low-cost electrochemical aptasensor by integrating nanoporous carbon derived from a zeolitic imidazolate framework-8 (ZIF-8) with methylene blue (MB) as a redox tag and an AuNP-modified GCE for signal readout (Figure 25). The ZIF-8 precursor, synthesized from 2-methylimidazole, was carbonized at different temperatures, with optimal performance achieved at 700 °C (ZC-700), which offered the best yield of stimuli-responsive nanoporous carbon. The morphology and structure of ZIF-8 and ZC-700 were thoroughly characterized by TGA, FESEM, TEM, XRD, and Raman spectroscopy, confirming successful carbonization with an ID/IG ratio of 1.12 (intensity ratio between the D peak at 1350 cm^−1^ and G peak at 1600 cm^−1^ of graphite). The sensing mechanism exploited π–π stacking to seal MB within ZC-700 using AβO-specific aptamers; in the presence of AβO, aptamers preferentially detached from MB to bind the target, releasing MB for electrochemical detection. MB released from ZC-700 was captured at the AuNP-modified GCE via hybridization with a DNA capture probe, generating a measurable DPV signal. This configuration combined the high surface area, suitable pore size, and post-carbonization stability of ZC-700 with a clever non-covalent aptamer-gating strategy. Under optimized conditions, the aptasensor achieved an ultra-low LOD of 1.58 fM over a wide linear range of 50 fM–10 nM, as determined by DPV and supported by EIS studies. The Apt/MB/ZC-700 sensor exhibited strong selectivity against four interfering species (Aβ monomers, Aβ fibrils, tau protein, and α-synuclein), reproducibility over five independent fabrications (RSD 3.42%), and stability over eight days (RSD < 3.5%). In human serum analyzed by the standard addition method, the device achieved recoveries of 102.35–107.14% with RSD 1.54–3.55%. The combination of nanoporous carbon’s loading capacity with aptamer-based signal gating offers a promising, low-cost approach for early AD diagnosis. However, further validation with clinical AD patient samples, extended stability studies, and benchmarking against established techniques such as SERS, SPR, or advanced ELISA formats are required to establish its practical diagnostic utility and commercial practicability.

Other carbon-based electrochemical biosensors for AD biomarker detection (Table 6) illustrate how diverse carbon allotropes and morphologies can be exploited for sensitive and selective analysis in complex biological matrices. Reported strategies include eco-friendly, low-cost paper-based electrodes incorporating molecularly imprinted polymer (MIP) layers, superhydrophobic carbon fiber composites decorated with gold–platinum alloy nanoparticles, and nanoporous carbons derived from metal–organic frameworks for aptamer-gated redox release. These platforms achieved detection limits from the femtomolar to the sub-ng/mL range, with wide linear ranges and strong selectivity, often validated in spiked human serum. Key advantages include low material cost, antifouling surfaces, and adaptable fabrication routes. However, limitations remain, including scarce long-term stability studies, limited reproducibility assessment, and the absence of clinical patient validation. Overall, these findings highlight the potential of alternative carbon nanomaterials in electrochemical biosensing, particularly when integrated with molecular recognition elements and scalable production methods.

## 4. Comparative Analysis of Recognition Elements and Targeted Biomarkers

The reviewed CNM-based electrochemical sensors for the detection of Alzheimer’s disease (AD) biomarkers employed three main types of recognition elements: aptamers, antibodies, and molecularly imprinted polymers (MIPs). Across all works in Section 3, aptamers were the most common (14 studies, ~58%), followed by antibodies (7 studies, ~29%) and MIPs (3 studies, ~13%).

Aptamers formed the majority of recognition elements ([90,122,123,124,125,130,132,134,137,138,140,141,143,144]), reflecting their adaptability, chemical stability, and high affinity in complex matrices. Notable examples include Zhou et al. (2021) [122], who achieved an LOD of 3.5 pM for Aβ oligomers in human serum using a dual-recognition aptasensor on vertical graphene scaffolds; Vajedi et al. (2024) [124], who reached 48.6 fg/mL for Aβ_1–42_ in plasma with a Ni–Co–P MOF/rGO/AuNP hybrid; and Liu et al. (2024) [134], who reported an aptamer-based EIS sensor for Aβ_1–42_ with excellent selectivity in serum. Ren et al. (2023) [144] also achieved an ultralow LOD of 1.58 fM for Aβ_1–42_ using nanoporous carbon with aptamer-gated methylene blue release. Li et al. (2025) [90] advanced this field by integrating a TiO_2_/Au-g-C_3_N_4_ heterojunction with an Aβ_40_-specific aptamer, enabling an ultralow LOD of 0.33 fg/mL and validation in diluted CSF and plasma. A key strength across these works is the relative ease of aptamer functionalization on CNM surfaces, achieved through well-established chemistries such as thiol–gold bonds [90,132], π–π stacking [124,144], or EDC/NHS coupling [134,137,138], which enables straightforward integration into diverse electrode architectures. However, challenges remain, such as multi-step immobilization procedures to maintain aptamer conformation [144] and the frequent absence of long-term stability evaluation. While these platforms consistently demonstrated high sensitivity and selectivity for targets such as Aβ_1–42_ [124,132,134], Aβ oligomers [122,123,125,137,138,141,143,144], Aβ_1–40_ [90,125,132,140], and tau protein [130], most studies that reported storage stability data included typically days to weeks [90,122,123,124,125,134,137,138,141,143,144], whereas others did not evaluate storage stability, leaving the question of extended shelf-life performance unanswered.

Antibody-based immunosensors ([6,119,120,121,131,133,136]) accounted for nearly one-third of the reviewed studies. They remain a clinically validated choice for AD biomarker detection due to their high affinity and established specificity. Karaboga and Sezgintürk (2020) [121] achieved a 0.091 pg/mL LOD for tau-441 in serum and CSF using rGO–AuNP electrodes, Sethi et al. (2020) [119] reported a 2.398 pM LOD for Aβ_1–42_ in plasma on a graphene/rGO dual-layer SPE, and Kim et al. (2020) [6] targeted multiple biomarkers, including Aβ_1–42_, Aβ_1–40_, t-tau, and p-tau181, in a CNT-based FET immunosensor. Schneider et al. (2022) [131] focused on p-tau181, while Gu et al. (2024) [133] incorporated Fetuin B alongside Aβ oligomers in a multiplexed format. Across these works, antibody-based platforms have consistently delivered robust selectivity and high recovery in real samples. However, antibody immobilization can be affected by the random orientation of the antibody molecule on the electrode surface, potentially reducing the availability of antigen-binding sites [119,131]. Regarding storage stability, four studies ([120,121,133,136]) evaluated storage performance at 4 °C, reporting short-term stabilities of up to 3 weeks, 10 weeks, 28 days, and 11 days, respectively, while the remaining antibody-based works ([6,119,131]) did not assess stability at all, leaving long-term performance unverified.

MIPs ([129,139,142]) were less common but provided strong examples of synthetic, low-cost recognition. Pereira et al. (2020) [142] employed a paper-based PEDOT–carbon ink electrode with an o-phenylenediamine MIP for Aβ_1–42_, achieving an LOD of 0.067 ng/mL at ~€0.03 per sensor. Pakapongpan et al. (2024) [139] combined a nitrogen-doped carbon dot–graphene nanohybrid with a polypyrrole MIP for Aβ_1–42_, reaching 1 pg/mL in artificial serum. Özcan et al. (2020) [129] used a CNT-based MIP for Aβ oligomers, demonstrating high stability and reproducibility. The main strengths observed in these works were their low production cost, physical and chemical robustness, and potential for regeneration and reuse [139,142]. However, in some cases, the imprinted polymer layers were inherently insulating, leading to reduced electron transfer efficiency unless conductive fillers or nanostructures were incorporated into the composite [139,142].

Table 7 presents the complete list of recognition elements from the reviewed works, along with their advantages, limitations, and targeted biomarkers.

From a biomarker perspective, amyloid-beta species were the most frequently targeted in the reviewed works. Aβ oligomers, including unspecified forms and AβO_42_, were detected in 9 studies (~38%), followed by Aβ_1–42_ in 7 studies (~29%) and Aβ_1–40_ in 5 studies (~21%). Tau proteins, comprising tau-441, total tau (t-tau), phosphorylated tau at threonine 181 (p-tau181), and unspecified tau protein, were targeted in 6 studies (~25%), often in combination with amyloid-beta to improve diagnostic accuracy. Fetuin B appeared in only one study (~4%) [133], included in a multiplexed assay alongside amyloid-beta oligomers.

Overall, aptamer-based platforms have predominated in recent CNM-based electrochemical sensors for AD biomarkers, supported by their chemical versatility, stability, and compatibility with nanostructure-enabled signal amplification. Antibody-based sensors remain the clinically recognized standard, particularly for tau-related biomarkers, while MIPs, although less common, offer a promising route to low-cost, durable, and reusable devices. The choice of recognition element should weigh not only analytical performance but also fabrication complexity, operational stability, and the intended diagnostic context.

## 5. Conclusions and Future Perspectives

Over the past five years, the integration of carbon nanomaterials (CNMs) into electrochemical biosensor architectures has enabled advanced, highly sensitive, and increasingly accessible platforms for the early detection of Alzheimer’s disease (AD) biomarkers. Novel designs have incorporated diverse CNM allotropes, such as graphene derivatives, carbon nanotubes, carbon dots, carbon nitride, and nanoporous carbons, combined with aptamers, antibodies, or molecularly imprinted polymers (MIPs). These approaches have delivered substantial gains in signal amplification, target selectivity, and antifouling properties, with several studies demonstrating robust performance in spiked or real biological samples and, in some cases, multiplex detection capabilities suitable for point-of-care (POC) formats. Selecting CNM types and morphologies according to the target analyte and sensing strategy has proven crucial for optimizing analytical performance.

Hybrid architectures that combine CNMs with metal nanoparticles, conductive polymers, or metal–organic frameworks have frequently outperformed single-component systems, benefiting from synergistic conductivity, catalytic, and adsorption properties. In parallel, detection modalities have diversified beyond classical voltammetry and impedance measurements to include electrochemiluminescence, photoelectrochemical, micromotor-driven, and paper-based formats, broadening the application scope of CNM-based biosensors.

Despite this progress, critical challenges remain. Multiplex assays for the simultaneous detection of multiple AD biomarkers are still scarce, and long-term stability studies are often limited to short storage periods. Reproducibility and batch-to-batch consistency are rarely addressed, and clinical validation using patient cohorts is still lacking. MIPs have emerged as promising low-cost, reusable recognition layers, but their insulating nature can hinder electron transfer in redox-probe-based methods such as DPV. Notably, CNM-enhanced paper-based platforms, micromotor systems, and densely aligned CNT films have shown encouraging analytical potential for multi-test clinical applications.

A quantitative assessment of CNM-based electrochemical sensors for AD detection from 2020 to mid-2025 reveals a predominance of aptamer-based platforms, valued for their adaptability, robustness in complex matrices, and compatibility with nanostructure-enabled amplification. Antibody-based sensors maintain a strong position, especially for tau protein detection, due to their clinically established specificity. MIP-based designs, though less common, offer advantages in durability and reusability. Amyloid-beta species, particularly oligomers and Aβ_1–42_, remain the primary targets, with tau proteins used to complement disease staging and differential diagnosis.

Looking ahead, future research should prioritize eco-friendly, cost-effective biosensor designs, favoring sustainable CNM substrates over heavy metals and other hazardous materials. The recent FDA authorization of the Lumipulse G pTau217/β-amyloid_1–42_ plasma assay marks a regulatory milestone for blood-based biomarker diagnostics and is likely to accelerate clinical translation of CNM-based electrochemical platforms. Enabling simultaneous, multi-biomarker detection in a single assay will be key to improving diagnostic accuracy, increasing robustness, and expanding the applicability of these sensors for early and reliable AD detection in POC settings. Equally important will be the development of scalable, reproducible fabrication routes that ensure consistency from laboratory prototypes to mass-produced clinical devices. Future work should also look at how CNM texture, orientation, and defect chemistry affect reproducibility and stability in biosensor interfaces.

## Figures and Tables

**Table 1:** Overview of diagnostic method classes for Alzheimer’s disease (AD).

Method Class	Technique (Examples)	Benefits	Drawbacks	References
Clinical/Cognitive	Clinical evaluation; neuropsychological testing	Widely used; accessible	Low accuracy and specificity in the early stages	[6,7]
Neuroimaging	MRI (structural changes); PET (amyloid, tau)	In vivo visualization of brain structure and pathology; accurate	Expensive; time-consuming; requires skilled personnel; limited accessibility	[5,6,7]
Fluid-based (invasive)	CSF analysis (Aβ, tau)	High accuracy; widely used biomarker detection method	Invasive lumbar puncture; costly; time-consuming; limited accessibility	[5,6,7]
Fluid-based (blood-based)	Plasma/serum assays (e.g., pTau217/Aβ_1–42_ ratio, CLEIA, Lumipulse G1200)	Less invasive; promising for early detection; FDA-authorized test; high sensitivity (~92%) and specificity (~97%)	Currently restricted to specialized labs; biomarker levels 10–100× lower than in CSF; interference from other plasma proteins	[6,8,9]
Spectroscopic/Analytical	Spectroscopy, chromatography, Raman spectroscopy	Multiplexed analysis; rapid; non-destructive	Low selectivity in complex matrices; high operational cost; requires specialized equipment and expertise	[5,10,11]

AD: Alzheimer’s disease; Aβ: Amyloid-beta; CSF: Cerebrospinal fluid; CLEIA: Chemiluminescent enzyme immunoassay; FDA: U.S. Food and Drug Administration; MRI: Magnetic Resonance Imaging; PET: Positron Emission Tomography; pTau: Phosphorylated tau protein.

**Table 2:** Summary of Graphene-based electrochemical biosensors for detection of Alzheimer’s disease biomarkers.

Reference	Detection Method	Detection Principle/Platform	LOD	Linear Range	Target/Sample Type	Real Samples
Sethi et al. (2020) [119]	DPV	Antibody (H31L21); external redox probe; signal-off (inhibition): Ab–Aβ_1–42_ binding hinders [Fe(CN)_6_]^3−^/^4−^ electron transfer (current decrease observed in CV and DPV upon Aβ_1–42_ binding); Platform: dual-layer graphene/rGO SPE with Pyr–NHS linker (π–π to rGO; NHS–amide to Ab); BSA blocking; CV used only for assembly/characterization	2.398 pM	11 pM–55 nM	Aβ_1–42_/PBS; Human plasma (spiked); mouse plasma	Yes—Human plasma (spiked), mouse plasma (no pretreatment); selectivity vs. Aβ_1–40_, ApoE ε4 (interferents 500 nM vs. 50 pM Aβ_1–42_, negligible). Incubation 60 min; IHC/MRI concordance (mouse)
Li et al. (2020) [120]	Amperometry (i–t)	Antibody; no external redox couple; signal-on (catalytic) via electrocatalytic H_2_O_2_ reduction at G/Co_9_S_8_–Pd/graphene; Ab–Aβ binding increases catalytic current (i–t readout); EIS (Rct increase) confirms electrode modification and antigen binding; Platform: G/Co_9_S_8_–Pd nanocomposite drop-cast on GCE; antibody immobilized via Pd–N coordination; BSA blocking; CV/EIS used only for assembly/characterization	41.4 fM	0.1 pg/mL–50 ng/mL	Aβ/PBS; Artificial CSF (spiked)	No—Artificial CSF (standard addition); recovery 96.3–109.5%; RSD 3.9–4.9%; selectivity vs. glucose, HIg, Gln, PSA; reproducibility RSD < 5% (*n* = 5); stability: ~4.1% signal loss after 3 weeks at 4 °C
Karaboga & Sezgintürk (2020) [121]	EIS, CV	Antibody (anti-Tau, monoclonal); external redox probe; signal-off (inhibition): Ab–Tau-441 binding hinders [Fe(CN)_6_]^3−^/^4−^ electron transfer (ΔRct increase, current decrease in CV); Platform: disposable ITO electrode coated with rGO–AuNP nanocomposite; anti-Tau immobilized via 11-MUA SAM + EDC/NHS coupling; BSA blocking; CV/EIS used for assembly/characterization	0.091 pg/mL	1–500 pg/mL	Tau-441/PBS; Human serum, CSF	Yes—Human serum (*n* = 5, standard addition) and human CSF (*n* = 4, standard addition); recovery 96.1–108.6%; repeatability RSD 6.38% (*n* = 20 electrodes); reproducibility RSD 3.02–3.41% (*n* = 6 batches); selectivity vs. HSP-70, α-Syn, RACK-1 (negligible); stability 8 weeks at 4 °C
Zhou et al. (2021) [122]	DPV	Aptamer-polyT-CuNP conjugates serve as signal tags, binding to AβO previously captured by PrP^C^ (95–110 residues); signal-on via poly(thymine)-templated CuNP tags (DPV peak from Cu^0^/Cu redox); double amplification by CuNP tags and Au-VG/CC substrate; Platform: Au-nanoparticle-modified vertical graphene on carbon cloth (Au-VG/CC); HS-PrP^C^ immobilized by Au–S; hexanethiol blocking; EIS/CV used only for assembly/characterization	3.5 pM	10–2200 pM	Aβ oligomers/buffer; Human serum	Yes—Human serum (*n* = 9; AD + controls); standard-addition recovery 93–116%, RSD < 10%; repeatability RSD 4.8% (*n* = 7); reproducibility RSD 6.7% (*n* = 6); ELISA correlation; selectivity vs. Aβ monomers, Aβ fibrils, insulin (1 μg/mL), TNF-α (500 pg/mL), CRP (100 μg/mL) (negligible); stability: 89.9% signal retained after 4 weeks at 4 °C
Gallo-Orive et al. (2024) [123]	SWV	Aptamer (thiolated, AβO_42_-specific); label-free signal-off via inhibition of [Fe(CN)_6_]^3−^/^4−^ redox probe (SWV cathodic current decreases upon AβO_42_ binding); Platform: catalytic micromotors (GO–AuNPs/Ni/PtNPs tubular MM) providing self-propulsion and enhanced mixing; aptamer immobilized via Au–S, BSA blocking; EIS with Ru(NH_3_)_6_^3+^/^2+^ used only for assembly/binding confirmation (Rct increase); CV used only for characterization	0.10 pg/mL	0.5–500 pg/mL	AβO_42_/Brain tissue, CSF, Human plasma	Yes—Brain tissue, CSF, human plasma (AD patients); 5 µL undiluted; recovery 94–102%; precision RSD < 8% (intra-/inter-assay); selectivity vs. Aβ_42_ monomer (negligible); validated against dot blot (MM faster, 5 min vs. >14 h); stability of MM–aptamer complexes 15 days at 4 °C
Vajedi et al. (2024) [124]	DPV	Aptamer (thiolated, Aβ_42_-specific); label-free inhibition of [Fe(CN)_6_]^3−^/^4−^ redox probe (DPV peak current decreases upon binding); Platform: Au@(Ni + Cu)TPyP MOF/rGO nanostructure on GE; aptamer immobilized via Au–S; MCH blocking; CV/EIS used only to confirm assembly and Aβ_42_ binding (Rct increase)	48.6 fg/mL	0.05 pg/mL–5.00 ng/mL	Aβ_42_/Human serum (spiked)	Yes—Human serum (10% diluted); recovery 95–104%; RSD 4.3% (spiked serum); reproducibility RSD 5.6% (*n* = 5 sensors); regeneration RSD 3.96% (bound), 2.27% (regenerated), 5 cycles; selectivity vs. Hb, HSA, BSA, HEP (≤1000×, negligible); stability 95.6% after 10 days at 4 °C.
Fan et al. (2025) [125]	DPV	Aptamer (Aβ_1–40_ oligomers, thiolated); signal-off via inhibition of [Fe(CN)_6_]^3−^/^4−^ redox probe (ΔI decrease upon binding); ternary PPy–rGO–Fe_2_O_3_ nanocomposite enhances conductivity and surface area; AuNPs provide Au–S anchoring for aptamer; BSA blocking; Platform: PPy/rGO–Fe_2_O_3_/AuNPs/GCE; CV/EIS used only for assembly confirmation	40 fM	0.1 pM–200 nM	AβO (Aβ_1–40_)/PBS; Artificial serum (spiked)	No—artificial serum (spiked); recovery 96.3–105%; reproducibility RSD < 4.4% (*n* = 5 electrodes); selectivity not studied; stability ≈ 90.2% signal retained after 14 days

Aβ: Amyloid-beta; Aβ_1–40_: Amyloid-beta peptide containing 40 amino acids; Aβ_1–42_: Amyloid-beta peptide containing 42 amino acids; Aβ_42_: Amyloid-beta peptide containing 42 amino acids; AβF: Amyloid-beta fibrils; AβM: Amyloid-beta monomers; AβO: Amyloid-beta oligomers; AβO_42_: Amyloid-beta oligomers containing 42 amino acids; AD: Alzheimer’s disease; ApoE ε4: Apolipoprotein E epsilon 4; AuNPs: Gold nanoparticles; BSA: Bovine serum albumin; CC: Carbon cloth; CRP: C-reactive protein; CSF: Cerebrospinal fluid; CuNPs: Copper nanoparticles; CV: Cyclic voltammetry; DPV: Differential pulse voltammetry; EIS: Electrochemical impedance spectroscopy; ELISA: Enzyme-linked immunosorbent assay; GCE: Glassy carbon electrode; GO: Graphene oxide; Gln: Glutamine; H31L21: Anti-Aβ_1–42_ monoclonal antibody clone H31L21; Hb: Hemoglobin; HEP: Haptoglobin; HIg: Human immunoglobulin; HSA: Human serum albumin; HSP-70: Heat shock protein 70; IHC: Immunohistochemistry; IgG: Immunoglobulin G; ITO–PET: Indium tin oxide–polyethylene terephthalate; LOD: Limit of detection; MOF: Metal–organic framework; MRI: Magnetic resonance imaging; Ni–Co–P MOF: Nickel–cobalt–porphyrin metal–organic framework; PPy: Polypyrrole; PrP^C^: Cellular prion protein; PSA: Prostate-specific antigen; Pyr–NHS: N-hydroxysuccinimide pyrrole linker; RACK-1: Receptor for activated C kinase 1; rGO: Reduced graphene oxide; Rct: Charge-transfer resistance; RSD: Relative standard deviation; SPE: Screen-printed electrode; SWV: Square wave voltammetry; SYN-α: Alpha-synuclein; TNF-α: Tumor necrosis factor alpha; VG: Vertical graphene.

**Table 3:** Summary of CNT-based electrochemical biosensors for detection of Alzheimer’s disease biomarkers.

Reference	Detection Method	Detection Principle/Platform	LOD	Linear Range	Target/Sample Type	Real Samples
Özcan et al. (2020) [129]	DPV	MIP (electropolymerized polypyrrole; template Aβ_1–42_); label-free signal-off via inhibition of [Fe(CN)_6_]^3−^/^4−^ redox probe (DPV peak decreases upon rebinding); template eluted with NaCl (1.0 M); Platform: GCE modified with d-Ti_3_C_2_T_x_ MXene/MWCNTs (3:1 (m/m)) composite on GCE, OPDA electropolymerized with Aβ_1–42_ template; CV/EIS used only for assembly/characterization (ΔEp, Rct changes)	0.3 fg/mL	1.0–100.0 fg/mL	Aβ_42_/PBS; Human plasma (spiked)	Yes—Spiked human plasma; recovery 99.99–100.04% (*n* = 6); repeatability RSD 0.11% (60 runs); reproducibility RSD 0.15% (20 electrodes); selectivity vs. Hb, HEP, BIL (low cross-response; k up to 33.33); agreement with LC–MS/MS (Wilcoxon, *p* > 0.05); reusability ≥ 30 cycles; stability 60 days (inter-day RSD 0.13%)
Kim et al. (2020) [6]	Chemiresistive (ΔR, CNT-FET)	Antibodies (anti-Aβ_42_, anti-Aβ_40_, anti-t-tau, anti-p-tau181) immobilized via carbodiimide coupling (EDC/sulfo-NHS) after UV–ozone carboxylation of CNTs; binding of antigens increases resistance (scattering centers in p-type SWCNTs; ΔR readout); multiplexed detection of 4 biomarkers; Platform: densely aligned SWCNT monolayer by Langmuir–Blodgett on Si/SiO_2_ with Cr/Au contacts; BSA/Tween blocking; CV used only for assembly/characterization	2.13 fM (Aβ_42_), 2.20 fM (Aβ_40_), 2.45 fM (t-tau), 2.72 fM (p-tau181)	~100–10^6^ fM	Aβ_42_, Aβ_40_, t-tau, p-tau181/Human plasma	Yes—Human plasma; spiked (recovery 93.0–97.6%); clinical plasma from AD patients and controls (*n* = 20 each); composite biomarkers (t-tau/Aβ_42_, p-tau181/Aβ_42_, Aβ_42_/Aβ_40_) discriminated AD vs. controls with 90.0% sensitivity, 90.0% selectivity, and 88.6% accuracy (AUC ≥ 0.93)
Yin et al. (2022) [130]	EIS	Aptamer (NH_2_-ended, tau-specific) immobilized on MWCNTs-modified electrode via APTES linker; NH_2_-PEG blocking; tau binding decreases charge-transfer resistance (ΔRct, Nyquist plots); Platform: MWCNT-modified electrode with APTES linker and aptamer immobilization; CV/EIS used only for assembly/characterization	1 fM	1 fM–1 nM	Tau protein/Human serum (spiked; 1:100 dilution)	Yes—Spiked human serum (1:100 dilution); selectivity confirmed vs. complementary aptamer, CFH, albumin (low-cross response)
Schneider et al. (2022) [131]	SWV	Antibody (anti-p-Tau181, polyclonal) randomly adsorbed (“flat-on”) on MWCNTs–PAH/Pt nanocomposite; label-free signal-off via inhibition of [Fe(CN)_6_]^3−^/^4−^ redox probe (SWV peak current decreases upon p-Tau181 binding); BSA blocking; Platform: pretreated carbon SPE (C-SPE) modified with MWCNTs–PAH/Pt nanocomposite for Ab anchoring; CV used only for assembly/characterization	0.24 pg/mL	8.6 pg/mL–1100 pg/mL	p-Tau181/FBS (1:100, 1:10)	No—FBS (spiked); recovery 87–95%; selectivity vs. IgG, Hb, uric acid, BSA (interference ≤ 8%)
Chen et al. (2022) [132]	FET	Aptamers (thiolated DNA; Aβ_42_, Aβ_40_) immobilized on AuNPs via Au–S; signal-off: aptamer conformational change upon binding causes Vth shift and IDS decrease; Platform: high-purity semiconducting CNT network channels on Si/SiO_2_ with 6 nm Y_2_O_3_ gate dielectric, AuNPs as floating-gate linkers; multi-blocking (MCH, Tween-20, BSA) to suppress nonspecific adsorption; wafer-scale device fabrication; Raman/SEM/fluorescence and electrical transfer curves used only for assembly/characterization	45 aM (Aβ_42_), 55 aM (Aβ_40_)	1 fM–10 pM	Aβ_42_, Aβ_40_/Human serum	Yes—Undiluted human serum (spiked); recovery 88–108%; selectivity vs. BSA, IgG, non-target Aβ peptide; selectivity ratios up to 800% (Aβ_42_), 730% (Aβ_40_); CV < 10%; stability 30 min in serum; response ~40 s
Gu et al. (2024) [133]	SWV	Antibodies (A11 for Aβ oligomers; anti-Fetuin B); in sandwich format with dmSiO_2_–Au–Thionine-Ab nanoprobes; ratiometric readout vs. ferrocene (I_Thi_/I_Fc_); Platform: multiplex paper-based electrode made by vacuum-filtered SWCNT underlayer + AuNPs (3 WEs + Au CE + Ag pseudo-RE); BSA blocking; CV used only for assembly confirmation	0.005 ng/mL (AβO), 0.02 ng/mL (Fetuin B)	0.01–40 ng/mL (AβO), 0.05–80 ng/mL (Fetuin B)	AβO (hippocampus, cortex, serum) and Fetuin B (serum)/APP/PS1 transgenic mice	Yes—APP/PS1 transgenic mouse hippocampus, cortex, and serum (AβO) and serum (Fetuin B); recovery 98.0–100.9% (AβO hippocampus), 98.9–109.8% (AβO serum), 97.7–106.7% (Fetuin B serum); selectivity vs. dopamine, ascorbic acid, amino acids (Val, Cys, Ser, Glu, Thr), ions (Na^+^, Fe^2+^, Ca^2+^, Cu^2+^); note: oligomeric Aβ_40_ gives similar signal to Aβ_42_ (A11 oligomer-specific); no cross-talk between channels; repeatability RSD 2.51% (AβO), 2.96% (Fetuin B); reproducibility RSD 4.97% (AβO), 4.28% (Fetuin B); stability: ~89% response retained at 28 days (AβO only)
Liu et al. (2024) [134]	SWV	Aptamer (amine-terminated Aβ_42_ ssDNA) covalently immobilized via EDC/NHS onto COOH–CNTs; label-free binding decreases interfacial resistance, measured as increased SWV current; BSA blocking; Platform: freestanding electrospun PA/PANI–CNTs nanofiber membrane electrode; CV/EIS with [Fe(CN)_6_]^3−^/^4−^ used only for electrode assembly confirmation	30 fg/mL	0.1 pg/mL–500 pg/mL and 500 pg/mL–110 ng/mL	Aβ_42_/Human serum	Yes—Human serum (spiked; protein-depleted); recovery 97.65–111.50%; RSD 0.09–7.00%; selectivity vs. alpha-fetoprotein, cTnI, CA125, hCG (100 ng/mL each); reproducibility RSD 0.96% (*n* = 6); electrochemical stability after 30 CV scans (−8.35%, −7.23% peak changes); 30-day storage stability; response time 4 min (72.6% signal at 2 min)

Aβ: Amyloid-beta; Aβ_40_: Amyloid-beta peptide containing 40 amino acids; Aβ_42_: Amyloid-beta peptide containing 42 amino acids; AβO: Amyloid-beta oligomers; Ab: Antibody; AFP: Alpha-fetoprotein; APTES: (3-Aminopropyl)triethoxysilane; APP/PS1: Amyloid precursor protein/presenilin-1 transgenic mouse model; Au CE: Gold counter electrode; AuNPs: Gold nanoparticles; BIL: Bilirubin; BSA: Bovine serum albumin; CA125: Cancer antigen 125; C-SPE: Carbon screen-printed electrode; CFH: Complement factor H; COOH–CNTs: Carboxyl-functionalized carbon nanotubes; CNT: Carbon nanotube; Cr/Au: Chromium/gold contacts; CV: Cyclic voltammetry; cTnI: Cardiac troponin I; ΔE_P_: Peak-to-peak separation; ΔRct: Change in charge-transfer resistance; d-Ti_3_C_2_T_X_ MXene: Delaminated titanium carbide MXene; dmSiO_2_–Au–Thi: Dendritic mesoporous silica nanoparticles–gold–thionine; DPV: Differential pulse voltammetry; EDC: N-(3-dimethylaminopropyl)-N′-ethylcarbodiimide; EIS: Electrochemical impedance spectroscopy; FBS: Fetal bovine serum; FET: Field-effect transistor; GCE: Glassy carbon electrode; hCG: Human chorionic gonadotropin; Hb: Hemoglobin; HEP: Heparin; IgG: Immunoglobulin G; LC–MS/MS: Liquid chromatography–tandem mass spectrometry; LOD: Limit of detection; MCH: 6-Mercapto-1-hexanol; MWCNTs: Multi-walled carbon nanotubes; NH_2_-PEG: Amine-terminated polyethylene glycol; PA: Polyamide; PAH: Poly(allylamine hydrochloride); PANI: Polyaniline; PBS: Phosphate-buffered saline; p-tau181: Tau protein phosphorylated at threonine 181; Rct: Charge-transfer resistance; RE: Reference electrode; RSD: Relative standard deviation; Si/SiO_2_: Silicon/silicon dioxide substrate; SPE: Screen-printed electrode; SWCNTs: Single-walled carbon nanotubes; SWV: Square wave voltammetry; t-tau: Total tau protein; Tween-20: Polyoxyethylene (20) sorbitan monolaurate; UA: Uric acid; UV–ozone: Ultraviolet ozone treatment; Vth: Threshold voltage; WE: Working electrode; Y_2_O_3_: Yttrium oxide dielectric.

**Table 4:** Summary of hybrid carbon-based (CNT/graphene) electrochemical biosensors for the detection of Alzheimer’s disease biomarkers.

Reference	Detection Method	Detection Principle/Platform	LOD	Linear Range	Target/Sample Type	Real Samples
Li et al. (2020) [136]	DPV	Antibody (anti-Tau-441); antigen–antibody complex blocks [Fe(CN)_6_]^3−^/^4−^ redox probe (DPV peak current decrease, ΔI); Platform: GE coated with MWCNTs–rGO–CS film; antibody immobilized via GLA; Tau-441–AuNPs conjugate for additional signal amplification; CV/EIS used for assembly confirmation	0.46 fM	0.5–80 fM	Tau-441 protein/Human serum	Yes—Human serum (clinical cohorts: 14 healthy, 14 MCI, 14 dementia); recovery 90.67–102.33%, repeatability RSD < 5%; reproducibility RSD 4.74% (*n* = 3); selectivity vs. Glu, AA, L-cys, α-Syn, HSA (<5% interference); stability 11 days at 4 °C (signal retained 92.86%)
Tao et al. (2021) [137]	DPV	Aptamer (AβO-specific); AβO–aptamer binding blocks [Fe(CN)_6_]^3−^/^4−^ redox probe (DPV ΔI decrease); Platform: GCE modified with Th-rGO-MWCNTs (3D nanocomposite); aptamer immobilized via EDC/NHS; BSA blocking; CV/EIS used only for assembly/characterization	10 fM	0.0443–443.00 pM	Aβ oligomers/Human serum (diluted)	Yes—Human serum (diluted); recovery 99.71–103.84%, repeatability RSD < 1% (0.79–0.98%); reproducibility RSD < 2% (*n* = 3 electrodes, 4.43 pM); selectivity vs. Aβ monomers, Aβ fibrils, α-syn oligomers, tau protein; stability 15 days at 4 °C (signal ≥ 90%), <90% on day 16; incubation time 20 min (optimized)
Negahdary et al. (2023) [138]	DPV	Aptamer (NH_2_-modified, AβO-specific); AβO–aptamer binding blocks [Fe(CN)_6_]^3−^/^4−^ redox probe (DPV peak decrease, ΔI); Platform: GCE with electrodeposited jagged Au (JG) nanostructures over-coated with GO-c-MWCNTs; aptamer immobilized via EDC/NHS; CV/EIS used only for assembly/characterization	0.088 pg/mL	0.1 pg/mL–1 ng/mL	Aβ oligomers/Human serum	Yes—Human serum (spiked; *n* = 10; diluted 50% in PBS): recovery 93–110% (overall RSD 5.43%); selectivity vs. AβMs, AβFs, and mixtures (max DPV decrement ≈ 38%); reproducibility RSD 1.28% (*n* = 5 re-fabrications); reversibility 3 cycles; stability 11 days under refrigerated storage (tracked every other day)
Pakapongpan et al. (2024) [139]	SWV	MIP (PPY, Aβ_42_ template); Aβ_42_ rebinding blocks [Fe(CN)_6_]^3−^/^4−^ redox probe (SWV peak decrease, ΔI); Platform: NCD–G nanohybrid-modified SPCE; PPY electropolymerized and film formation/template removal confirmed by CV; template removed with oxalic acid	1 pg/mL	5–70 pg/mL	Aβ_42/_artificial serum	No—Artificial serum (spiked): recovery 92.31–119.25%; repeatability RSD ≤ 5.44% (*n* = 3); reproducibility RSD 2.08% (*n* = 15 electrodes); selectivity vs. BNP, IgG, HSA; rebinding time 10 min (optimized)

AA: Ascorbic acid; Aβ: Amyloid-beta; Aβ_1–42_ (Aβ_42_): Amyloid-beta peptide containing 42 amino acids; AβO: Amyloid-beta oligomers; ABFs: Amyloid-beta fibrils; ABMs: Amyloid-beta monomers; α-Syn: Alpha-synuclein; AuNPs: Gold nanoparticles; BNP: B-type natriuretic peptide; BSA: Bovine serum albumin; CS: Chitosan; CV: Cyclic voltammetry; DPV: Differential pulse voltammetry; EDC: 1-ethyl-3-(3-dimethylaminopropyl)carbodiimide; EDC/NHS: 1-ethyl-3-(3-dimethylaminopropyl)carbodiimide/N-hydroxysuccinimide; GCE: Glassy carbon electrode; GE: Gold electrode; GLA: Glutaraldehyde; GO-c-MWCNTs: Graphene oxide–carboxylated multi-walled carbon nanotubes; HSA: Human serum albumin; JG: Jagged gold nanostructure; L-cys: L-cysteine; LOD: Limit of detection; MCI: Mild cognitive impairment; MIP: Molecularly imprinted polymer; MMSE: Mini-Mental State Examination; MWCNTs: Multi-walled carbon nanotubes; NCD–G: Nitrogen-doped carbon dot–graphene; PPY: Polypyrrole; RSD: Relative standard deviation; rGO: Reduced graphene oxide; SPCE: Screen-printed carbon electrode; SWV: Square wave voltammetry; Th: Thionine.

**Table 5:** Summary of g-C_3_N_4_-based electrochemical and photoelectrochemical biosensors for the detection of Alzheimer’s disease biomarkers.

Reference	Detection Method	Detection Principle/Platform	LOD	Linear Range	Target/Sample Type	Real Samples
Zhang et al. (2020) [140]	ECL	Aptamer (Aβ-targeting, thiolated); label-free signal-ON aptasensor; g-C_3_N_4_–heme nanocomposite as ECL luminophore; heme catalyzes in situ H_2_O_2_ production from Aβ–O_2_ interaction; K_2_S_2_O_8_ as co-reactant (dual catalytic ECL amplification); readout ΔI_ECL_; Platform: GE with aptamer immobilized via Au–S; BSA blocking; followed by incubation with Aβ and g-C_3_N_4_–heme; CV/EIS used only for assembly confirmation	3.25 fM	10 fM–0.1 μM	Aβ_40_ monomer/Human serum (spiked)	Yes—human serum (healthy donors); standard-addition recovery 95.3–104.1%; reproducibility RSD 4.65% at 10 nM (*n* = 5 electrodes); selectivity vs. Aβ_40_ oligomers, Aβ_40_ fibrils, BSA, CEA, thrombin; stability: no significant loss over 200 s consecutive scans; incubation 12 h at 37 °C
Zhang et al. (2021) [141]	PEC (cathodic)	Aptamer; on–off–on cathodic PEC aptasensor; p–n heterojunction CuO/g-C_3_N_4_ photocathode with aptamer-labelled MoS_2_ QDs@Cu NWs as dual amplifier (PEC enhancer + nanozyme for 4-CN/H_2_O_2_ precipitation); photocurrent decrease via insulating 4-CD and recovery upon AβO binding; readout ΔI_PEC_; Platform: ITO with CuO/g-C_3_N_4_, cDNA, BSA, MoS_2_ QDs@Cu NWs–aptamer; EIS used only for assembly confirmation	5.79 fM	10 fM–0.5 μM	Aβ oligomers/Human serum (spiked, 50-fold dilution)	Yes—Human serum (two samples; standard addition); recovery 98.20–103.12%, RSD 3.31–3.49% (two serum samples); reproducibility RSD 3.35% (*n* = 6 sensors); selectivity vs. AβM, AβF, TNF-α, Lys, Ins (10×, negligible response); stability: 10 on/off cycles ≈101.13% of initial; long-term storage 14 days at 4 °C ≈ 91.69% signal retained
Li et al. (2025) [90]	PEC	Aptamer; signal-on PEC aptasensor; TiO_2_/Au-g-C_3_N_4_ heterojunction on FTO (TiO_2_ by EPD + annealing, Au–C_3_N_4_ drop-coated); AuNPs enhance conductivity, LSPR, and provide Au–S sites for aptamer immobilization; thiolated Aβ_40_ aptamer immobilized via Au–S, MCH blocking; photocurrent increase upon Aβ_40_ binding; readout ΔI_PEC_; no external redox probe used	0.33 fg/mL	10^−15^–10^−11^ g/mL	Aβ_40_/PBS, CSF, plasma, artificial saliva	Yes—Clinical CSF (*n* = 3) and plasma (*n* = 6) vs. SiMoA; reproducibility RSD 2.69% (*n* = 6 sensors); RSD ≤ 5.9% in patient samples; selectivity vs. tau, Aβ_42_, AA, glucose, urea, chitosan (1000×); stability: 9 days at RT, ≈ 6.9% signal loss

4-CN: 4-chloro-1-naphthol; 4-CD: Benzo-4-chlorohexadienone (oxidation product of 4-CN); AA: Ascorbic acid; Aβ: Amyloid-beta; Aβ40: Amyloid-beta peptide containing 40 amino acids; Aβ42: Amyloid-beta peptide containing 42 amino acids; AβO: Amyloid-beta oligomers; AuNPs: Gold nanoparticles; BSA: Bovine serum albumin; CEA: Carcinoembryonic antigen; CSF: Cerebrospinal fluid; CV: Cyclic voltammetry; ECL: Electrochemiluminescence; EIS: Electrochemical impedance spectroscopy; FTO: Fluorine-doped tin oxide; g-C_3_N_4_: Graphitic carbon nitride; Glu: Glucose; Ins: Insulin; ITO: Indium tin oxide; Lys: Lysozyme; LOD: Limit of detection; MCH: 6-mercapto-1-hexanol; MoS_2_ QDs@Cu NWs: Molybdenum disulfide quantum dots-decorated copper nanowires; PEC: Photoelectrochemical; RSD: Relative standard deviation; RT: Room temperature; SiMoA: Single-molecule array; Tau: Tau protein; Thrombin: Coagulation factor II; TNF-α: Tumor necrosis factor alpha.

**Table 6:** Summary of other carbon-based electrochemical biosensors for the detection of Alzheimer’s disease biomarkers.

Reference	Detection Method	Detection Principle/Platform	LOD	Linear Range	Target/Sample Type	Real Samples
Pereira et al. (2020) [142]	SWV	MIP (template Aβ_42_; polymer OPDA); signal-off rebinding blocks [Fe(CN)_6_]^3−^/^4−^; readout ΔI; Platform: paper-based carbon-ink electrode (CI-HME) coated with PEDOT and ATP linker; OPDA electropolymerized with Aβ_42_; template removed by trypsin + oxalic acid; CV/EIS used only for assembly confirmation	0.067 ng/mL	0.1 ng/mL–1 μg/mL	Aβ_42_/PBS; serum (FBS, spiked)	Yes—serum (FBS, Cormay, spiked); recovery not reported; repeatability RSD < 10%; detection time 20 min; selectivity vs. BSA (4 mg/mL), glucose (0.7 mg/mL), creatinine (1 μg/mL); low-cost (~€0.03/sensor)
Liu et al. (2021) [143]	DPV	Aptamer; signal-off DPV with Fc redox probe; superhydrophobic CFP/AuPt nanoalloy boosts area and resists fouling; thiolated AβO aptamer self-assembled on AuPt; optional BSA blocking; readout ΔI (Fc); Platform: CFP/AuPt electrode with aptamer and BSA (optional); CV used only for characterization	0.16 pg/mL	0.5–10,000 pg/mL	AβO/PBS; Human serum (spiked)	Yes—human serum (spiked 1–1000 pg/mL); LOD 0.21 pg/mL (PBS with BSA), 0.90 pg/mL (serum); recovery 92.5–109% (*n* = 5), RSD < 10%; selectivity vs. Aβ_40_, Aβ_42_, Tau441, NFL, HSA (each 1 μg/mL, 1000× of AβO at 1 ng/mL); antifouling: ≈90% current retained after 168 h in serum; stability: sensor maintained performance for 60 days
Ren et al. (2023) [144]	DPV	Aptamer; nanoporous carbon from ZIF-8 (ZC-700) loaded with methylene blue and sealed by AβO aptamers; (π–π stacking, stimuli-responsive signal-ON); AβO binding opens gate, MB released and hybrid-captured on AuNP-modified GCE for amplification; readout ΔI; Platform: AuNP-coated GCE with thiolated capture probe (Au–S) + MCH blocking; ZC-700@MB/aptamer used as solution-phase nanocontainer; CV/EIS used only for assembly confirmation	1.58 fM	50 fM–10 nM	AβO/PBS; Human serum (spiked, 10× dilution)	Yes—human serum (standard addition, 10×); recovery 102.35–107.14%, RSD 1.54–3.55%; reproducibility RSD 3.42% (*n* = 5); selectivity vs. AβM, AβF, α-Syn, tau (10 nM interferents vs. 1 nM AβO, negligible); stability 8 days at 4 °C, signal retention ≈98.6–104.4%

Aβ: Amyloid-beta; Aβ_1–40_ (Aβ_40_): Amyloid-beta peptide containing 40 amino acids; Aβ_1–42_ (Aβ_42_): Amyloid-beta peptide containing 42 amino acids; AβF: Amyloid-beta fibrils; AβM: Amyloid-beta monomers; AβO: Amyloid-beta oligomers; α-Syn: Alpha-synuclein; AuNP: Gold nanoparticle; AuPt: Gold–platinum alloy; BSA: Bovine serum albumin; CFP: Carbon fiber paper; CI-HME: Carbon-ink hand-made electrode; CV: Cyclic voltammetry; DPV: Differential pulse voltammetry; EIS: Electrochemical impedance spectroscopy; Fc: Ferrocene; FBS: Fetal bovine serum; GCE: Glassy carbon electrode; HSA: Human serum albumin; MB: Methylene blue; MCH: 6-mercapto-1-hexanol; MIP: Molecularly imprinted polymer; NFL: Neurofilament light protein; OPDA: o-Phenylenediamine; PEDOT: Poly(3,4-ethylenedioxythiophene); RSD: Relative standard deviation; SWV: Square wave voltammetry; Tau441: Tau protein containing 441 amino acids; ZC-700: ZIF-8–derived nanoporous carbon carbonized at 700 °C; ZIF-8: Zeolitic imidazolate framework-8.

**Table 7:** Summary of recognition elements in CNM-based electrochemical biosensors for AD biomarkers.

Recognition Element	Advantages	Limitations	Biomarkers
Aptamers [90,122,123,124,125,130,132,134,137,138,140,141,143,144]	High specificity and selectivity; chemical stability; easy functionalization; effective in complex matrices	Multi-step immobilization required to preserve binding; limited long-term stability data in some cases	Aβ_1–42_ [124,132,134]; Aβ oligomers [122,123,125,137,138,141,143,144]; Aβ_1–40_ [90,125,132,140]; tau protein [130]
Antibodies [6,119,120,121,131,133,136]	Clinically validated specificity; high affinity; robust selectivity in serum/plasma	Orientation effects can reduce capture efficiency; stability under extended storage not always assessed	Aβ_1–42_ [6,119]; Aβ_1–40_ [6]; Aβ (unspecified) [120]; tau-441 [121,136]; t-tau [6]; p-tau181 [6,131]; Fetuin B [133]
MIPs [129,139,142]	Low cost; physical/chemical robustness; potential for reuse	Possible lower selectivity for closely related isoforms; insulating layers may hinder electron transfer	Aβ_1–42_ [139,142]; Aβ oligomers [129]
